# Structural and functional investigation of DinG containing a 3′–5′ exonuclease domain

**DOI:** 10.1128/mbio.00884-25

**Published:** 2025-06-30

**Authors:** Tianwen Gao, Wanshan Hao, Jin Gao, Yiyang Sun, Yukang Sun, Jieyu Yang, Kaiying Cheng

**Affiliations:** 1 Zhejiang Key Laboratory of Medical Epigenetics, Department of Immunology and Pathogen Biology, School of Basic Medical Sciences, Affiliated Hospital of Hangzhou Normal University, Hangzhou Normal University 356964, Hangzhou, Zhejiang, China; 2 State Key Laboratory for Diagnosis and Treatment of Infectious Diseases, The First Affiliated Hospital, College of Medicine, Zhejiang University 12377, Hangzhou, Zhejiang, China; Duke University School of Medicine, Durham, North Carolina, USA

**Keywords:** DinG, helicase, exonuclease, protein structure, MMC

## Abstract

Damage-inducible gene G (DinG), a bacterial homolog of SF2 helicase, has been extensively studied in *Escherichia coli*. However, the structural and functional characteristics of DinG homologs fused with an N-terminal 3′–5′ exonuclease domain, such as *Staphylococcus aureus* DinG (SaDinG), remain unexplored. In this study, we demonstrate that SaDinG possesses 3′–5′ exonuclease activity and exhibits 5′–3′ helicase activity on diverse DNA substrates, including splayed duplexes, 5′-overhangs, double flaps, bubbles, and gapped duplexes, resolving prior ambiguities about its biochemical functions. Intriguingly, both enzymatic activities were inhibited by elevated ATP concentrations, suggesting a potential ATP-dependent regulatory mechanism *in vivo*. We determined the crystal structures of SaDinG bound to ssDNA at ~3.2 Å resolution and identified key residues essential for its helicase and exonuclease activities through mutational analysis. Phenotypic studies revealed that a SaDinG deletion mutant exhibited heightened sensitivity to DNA crosslinking agents (mitomycin C and formaldehyde) but retained wild-type susceptibility to other DNA-damaging compounds. Complementation with either nuclease-dead or helicase-dead variants failed to restore crosslink resistance, indicating that both activities are indispensable for DNA crosslink repair. These results support a model in which SaDinG functions as a coordinated nuclease-helicase machine specifically adapted for DNA crosslink repair, with its dual enzymatic activities being tightly regulated by physiological ATP concentrations.

**IMPORTANCE:**

DNA helicases and exonucleases play essential roles in genome maintenance; however, little is known about bacterial helicase-exonuclease fusion proteins. This study examines DNA helicases and exonucleases that play essential roles in genome maintenance; however, little is known about bacterial helicase-exonuclease fusion proteins. This study provides the first structural and functional characterization of *Staphylococcus aureus* DinG (SaDinG), a unique enzyme that combines 5′-3′ helicase and 3′-5′ exonuclease activities. Our findings resolve previous uncertainties about SaDinG's function and reveal an ATP-dependent regulatory mechanism that modulates its activity. Additionally, we demonstrate that SaDinG is critical for bacterial resistance to DNA crosslinking agents. These insights not only expand our understanding of bacterial DNA repair but also suggest potential avenues for targeting DinG-like enzymes in antimicrobial strategies. Given the growing concerns over antibiotic resistance, understanding how bacteria maintain genome integrity under stress conditions is crucial. This work lays the foundation for further exploration of bacterial helicase-exonuclease systems and their role in genome stability and adaptive survival.

## INTRODUCTION

The xeroderma pigmentosum complementation group D/ damage-inducible G (XPD/DinG) helicase family, a subset of the Superfamily 2 (SF2) DNA helicases, translocates on single-stranded DNA (ssDNA) in the 5′–3′ direction and is conserved across all three domains of life (1, 2). *Escherichia coli* DinG (EcDinG), the most extensively studied DinG, was initially identified as a DNA damage-inducible gene in the SOS response following mitomycin C (MMC) treatment (3). Biochemical studies have shown that EcDinG unwinds diverse DNA structures, including overhangs, flap structures, forks, D-loops, R-loops, and G-quadruplex (G4) DNA (4–7). Additionally, EcDinG interacts with *E. coli* single-stranded DNA-binding protein (EcSSB), forming a stable complex that enhances helicase activity (8, 9). Another DinG-like homolog, YoaA, is also induced by DNA damage and interacts physically with the chi subunit (HolC) of the DNA polymerase III holoenzyme, likely through its 18 unique C-terminal residues. The YoaA-HolC complex exhibits DNA-dependent ATPase activity and preferentially unwinds forked duplex DNA with both 3′ and 5′ overhangs (10–12).

DinG homologs have also been identified in type IV CRISPR systems, termed CRISPR-associated DinG (CasDinG) (13). These proteins function as 5′–3′ helicases for double-stranded DNA (dsDNA) and RNA/DNA hybrids, playing essential roles in CRISPR interference by coordinating with other Cas proteins (14, 15). Deletion of *casdinG* or mutation of its ATP-binding site (K136A) abolishes interference in *Pseudomonas* (13, 16). A recently identified type IV system in *Sulfitobacter sp*. JL08 harbors a unique CasDinG variant with an additional C-terminal HNH nuclease domain (SulCasDinG-HNH), which is essential for RNA-guided, protospacer-adjacent motif (PAM)-dependent dsDNA degradation (17).

Structural analyses of XPD and EcDinG revealed a conserved four-domain architecture, comprising two helicase motor domains (MD1 and MD2) and two accessory domains: the Arch domain and the iron–sulfur (Fe-S) domain that coordinates a [4Fe-4S] cluster (14, 15, 18–23). The FeS domain and Arch domain are inserted into MD1 and critical for duplex DNA unwinding in XPD and EcDinG (5, 23, 24). Structural studies of EcDinG in complex with ssDNA and an ATP analog (ADP·BeF₃) support an “inchworm” mechanism for 5′–3′ translocation (23). The structure of PaCasDinG in complex with ssDNA displays the same DNA binding mode as EcDinG, indicating a similar ssDNA translocation mechanism (14).

Some DinG-like proteins possess an additional N-terminal exonuclease domain, although its structural details and precise biological function remain unclear (2). Unlike EcDinG, this variant is not regulated by LexA and may not participate in the SOS response in *Staphylococcus aureus* and *Bacillus subtilis* (25, 26). The most well-characterized example, *S. aureus* DinG (SaDinG), functions as an active 3′–5′ exonuclease on ssDNA and RNA substrates (27). Notably, no helicase activity has been reported for SaDinG, likely due to the absence of a complete FeS domain (27). However, *Pseudomonas aeruginosa* CasDinG (PaCasDinG) also lacks a fully conserved Fe-S domain yet retains strong helicase activity (14, 17).

In this study, we purified SaDinG and determined its ssDNA-bound complex structures using X-ray crystallography. We conducted a comprehensive investigation of its exonuclease activity and further confirmed its helicase activity on splayed duplex, gapped duplex, double-flap, bubble, and 5′-overhang substrates. Key residues responsible for both nuclease and helicase activities were identified through site-directed mutagenesis. Additionally, phenotype analysis of a SaDinG deletion mutant and various complementation strains revealed that SaDinG contributes to resistance to DNA crosslinking agents.

## MATERIALS AND METHODS

### Cloning and site-directed mutagenesis

All of the primers and oligonucleotides used in this study were purchased from Generay Biotech (Shanghai, China) and were listed in Table S1.

To construct the expression vectors, the full-length gene encoding SaDinG was amplified from *S. aureus* RN4220 genomic DNA by PCR and cloned into a modified pET28a expression vector, pET28T, which contains a fused N-terminal 6 × His tag and a TEV protease recognition site. Vectors were transformed into *E. coli* DH5α strain (TransGen Biotech, Beijing), selected from kanamycin-containing (50 µg/mL) plates, and further confirmed by sequencing.

Site-directed mutagenesis was performed with a QuikChangeTM Site-Directed Mutagenesis Kit from Stratagene (La Jolla, CA) as described previously (28). The fidelity of the constructed vectors was confirmed by sequencing. All the successfully constructed expression vectors were transformed into *E. coli* Rosetta (DE3) strain (TransGen Biotech, Beijing) for protein expression.

### Mutant strain construction and complementation

The *sadinG* deletion mutant was constructed by a pKOR1-mediated allelic replacement method (29). The upstream and downstream fragments of the target gene were amplified by PCR and then inserted into the *E. coli*/*S. aureus* shuttle vector, pKOR1. This vector allows for efficient cloning through lambda recombination and *ccdB* selection. The recombined pKOR1 plasmid was electroporated into *S. aureus* RN4220. The cells were grown at 43°C, a non-permissive condition for pKOR1 replication, which promotes homologous recombination and integration of the target fragment into the bacterial chromosome. Anhydrotetracycline-mediated induction of pKOR1-encoded *secY* antisense transcripts, a condition incompatible with staphylococcal growth, was utilized to select for chromosomal excision and plasmid loss. The mutant strain was complemented by introducing the pTX vector, which was ligated with either the wild-type *sadinG* gene or the mutated/truncated genes (via BamHI and MluI sites), into the deletion mutant, following the previously reported method (30). Also, the plasmid pTX16 (the empty vector) was introduced into the RN4220 strain as a control.

### DNA-damaging agent treatment assays

DNA-damaging agent treatment assays were conducted as previously described (31–34). Cells were grown in TSB medium with appropriate antibiotics until they reached the early exponential phase (OD_600_ = 1.0). For UV treatment, the cells were diluted to suitable concentrations and dotted on TSB agar plates. After complete absorption, the plates were exposed to UV at a dose of 100 J/m^2^. For resveratrol, MMC, and formaldehyde treatment, cells were first diluted to appropriate concentrations and dotted on TSB agar plates supplemented with either 100 µg/mL of resveratrol, or 8 µg/mL of MMC, or 0.4 mM of formaldehyde. In the case of Methyl-methanesulfonate (MMS) treatment, cells were treated with 10 mM of MMS for 15 min, then washed with PBS buffer, serially diluted 10-fold at each step, and dotted onto TSB agar plates. All plates were incubated at 30°C for 2–3 days before colonies were counted.

### Protein expression and purification

The protein expression and purification of tag-free SaDinG and mutants were conducted according to reference (28), with some modifications. In brief, transformed *E. coli* Rosetta (DE3) clones were grown at 37°C to an optical density of OD_600_ (0.8) in LB medium containing 50 µg/mL kanamycin. Protein expression was induced at 18°C for 18 h by adding isopropyl-b-D-thiogalactopyranoside (IPTG) at a final concentration of 0.4 mM. After harvesting, cells were re-suspended in lysis buffer (20 mM Tris [pH 7.5], 1 M NaCl, 5% (wt/vol) glycerol, 1 mM Tris [2-carboxyethyl] phosphine [TCEP], and 1 mM Ethylene Diamine Tetraacetic Acid [EDTA]), lysed by sonication, and centrifuged at 15,000 × *g* for 30 min at 4°C. The supernatant was purified using a cOmplete His-Tag Purification Column (Roche, Switzerland), which was equilibrated with buffer A (20 mM Tris [pH 7.5], 1 M NaCl, 5% (wt/vol) glycerol, 0.5 mM TCEP, and 1 mM EDTA), washed with 1 mM imidazole and finally eluted with 300 mM imidazole. The eluted fractions were subsequently diluted into 200 mM NaCl and loaded onto a Heparin HR column (Cytiva) pre-equilibrated with buffer B (20 mM Tris [pH 7.5], 200 mM NaCl, 1 mM EDTA, 5% (wt/vol) glycerol, and 1 mM TCEP). Fractions containing tagged SaDinG protein were eluted using a linear gradient of 0.2-1 M NaCl. After TEV-tag removal using TEV protease, the protein was reloaded onto the cOmplete His-Tag Purification Column to remove the uncleaved protein and TEV protease. The flow-through fractions were collected and concentrated. Proteins were finally purified by Superdex 200 10/300 Gl column (Cytiva) using buffer A.

Each fraction was analyzed by 12% sodium dodecyl sulfate polyacrylamide gel electrophoresis (SDS-PAGE). Fractions containing the purified proteins were pooled, concentrated, flash-frozen in liquid nitrogen, and stored at −80°C.

### Crystallization and structure determination

Crystallization trials were performed by the sitting drop vapor diffusion method at 289 K. In brief, the freshly purified protein was concentrated to about 20 mg/mL and centrifuged to remove insoluble fractions before crystallization. To create the SaDinG-ssDNA complex, 22 nt ssDNA was mixed with SaDinG in a 1.5:1 molar ratio. After a series of screening tests and optimizations, crystals of SaDinG-ssDNA complex were obtained from two different conditions. Crystals of SaDinG-ssDNA were obtained from 0.2 M LiSO_4_, 0.1 M MES (pH 6.5), and 15% Polyethylene glycol 4000. Crystals of SaDinG-ssDNA-Ca^2+^ were obtained in conditions of 5% vol/vol Tacsimate (pH 7.0), 0.1 M HEPES (pH 7.0), 8% wt/vol Polyethylene glycol monomethyl ether 5,000, and 50 mM CaCl_2_. Cryocooling was achieved by stepwise soaking of the crystals in a reservoir solution containing 10%, 20%, and 30% (wt/vol) glycerol for 1  min and flash freezing in liquid nitrogen. X-ray diffraction data were collected on beamline BL02U1 at Shanghai Synchrotron Radiation Facility (Shanghai, China) and integrated and scaled by Aquarium pipeline (35, 36). The structures of both complexes were determined using the molecular replacement method, with the *E. coli* DinG-ssDNA complex structure (PDB code: 6FWR) serving as the search model. Structures were refined using PHENIX (37) and interspersed with manual model building using COOT (38). All residues were in the most favorable and allowed regions of the Ramachandran plot. All structural figures were created by PyMOL. The statistics for the data collection and refinement are listed in Table 1.

**TABLE 1:** Statistics from crystallographic analysis^*a*^^,^^*b*^^,^^*c*^

Complex	SaDinG-ssDNA-Ca^2+^	SaDinG-ssDNA
PDB code	8ZEF	9II8
Data collection		
Source	BL02U1	BL02U1
Wavelength (Å)	0.9792	0.9790
Resolution (Å)	39.65-3.21 (3.33–3.21)	111.77-3.16 (3.27–3.16)
Space group	C222_1_	I,422
Cell dimensions: a, b, c (Å) α, β, γ (°)	68.86, 128.04, 303 90, 90, 90	229.17, 229.17, 128.03 90, 90, 90
Observation	300136 (46311)	319,824 (31982)
Unique reflections	22275 (3207)	29,417 (2913)
R_merge_ (%)	10.2 (51.4)	5.5 (55.1)
I/σI	17.3 (2.7)	10.6 (1.89)
Completeness (%)	99.9 (99.8)	100 (100)
Redundancy	13.4	8.8
Refinement statistics		
Resolution (Å)	39.65-3.21 (3.33–3.21)	111.77-3.16 (3.27–3.16)
R_factor_ (%)/R_free_ (%)	28.33/29.17	26.87/29.30
rmsd bonds (Å)/angles (°)	0.008/1.155	0.011/1.376
Ramachandran plot: Favored (%)/Outliers (%)	93.76/0	94.63/0

^*a*^ The numbers in parentheses refer to the outer shell.

^*b*^ R_factor_ = Σ||F(obs)- F(calc)||/Σ|F(obs)|.

^*c*^ R_free_ = R factor calculated using 5.0% of the reflection data randomly chosen and omitted from the start of refinement.

### Nuclease activity assays

Nuclease activity assays were conducted according to reference (34), with some modifications. All oligonucleotides were obtained from Generay (Shanghai, China) and labeled with 6-carboxfluorescein (6-FAM) at the 3′ or 5′ ends. The oligonucleotide sequences are provided in Table S1. For a typical nuclease assay, 100 nM of substrate was incubated with varying concentrations of freshly prepared wild-type or mutated SaDinG proteins in a 10 µL reaction volume containing 50 mM Tris (pH 7.5), 100 mM NaCl, 0.1 mg/mL BSA, 1 mM DTT, 5% (vol/vol) glycerol, and 1 mM MnCl_2_ at 37°C for 30 min. The reactions were stopped with 2 × stop buffer (10 mM EDTA, 98% formamide) and incubated at 100°C for 10 min, followed by rapid cooling on ice. Reaction products were resolved on 15% polyacrylamide gels containing 7 M urea. Gels were imaged in fluorescence mode (FAM) using ChemiScope6100 (Clinx Science Instruments, Shanghai). To assess metal preference, 100 nM 20 nt 5′ FAM-labeled poly dT was incubated with 10 or 100 nM SaDinG, in the presence of 10 mM of MgCl_2_, MnCl_2_, CaCl_2_, ZnCl_2_, NiCl_2_, or CoCl_2_. For the divalent metal ion concentrations test, 100 nM 20 nt 5′ FAM labeled poly dT was incubated with 5 nM SaDinG (when MnCl_2_ or CoCl_2_ was used), or 10 nM SaDinG (when MgCl_2_, NiCl_2_, or ZnCl_2_). Different concentrations (0, 0.313, 0.625, 1.25, 2.5, 5.0, and 10.0 mM) of divalent metal cations were introduced. For the determination of optimum substrate sequence, 0.8 mM substrates (5 nt poly dA, dT, dC, or dG) were incubated with 0.5 µM SaDinG and 10 mM Mn^2+^ for various durations (0, 5, 10, 15, 20, and 30 min), and the reaction products were subsequently separated using thin-layer chromatography (TLC), as described previously (34). The digestion fractions were calculated by Image J Software (National Institutes of Health, USA) from three replicates and displayed as line charts or columns using GraphPad Prism 9 (San Diego, USA).

### Real-time helicase activity assays

Real-time helicase activity assays were conducted by monitoring the increased fluorescent signals. Nine distinct substrates were designed for these assays (splayed duplex, annealed by Jb and Ja2; 3′-overhang, annealed by Jb and Ja; 5′-overhang, annealed by Jb2 and Ja2; double-flap, annealed by Jb, Ja2, and Jc3; holiday junction, annealed by Jb, Ja2, Jc3, and Jd; duplex, annealed by Jb and anti-Jb; bubble, annealed by J98 and J98b; gapped duplex 1, annealed by J98, J98b2, and anti-J98d; gapped duplex 2, annealed by J98u, J98d, and J98b). The sequences of the substrates are shown in Table S1. Each substrate encompassed an arm with a fluorescent (FAM) or quenching group (Dabsyl) labeled at each strand end of the duplex. Consequently, the unwinding of this arm could be quantified by observing the heightened fluorescent signals. Typically, 25 nM of substrate was incubated with either 500 nM of mutated DinG in a 20 µL reaction mixture containing 50 mM Tris-HCl (pH 8.0), 50 mM NaCl, 1 mM MgCl_2_, 100 µg/mL bovine serum albumin, 0.5 mM TCEP, and 5% glycerol. 1.0 mM ATP was introduced at the final step to initiate the reaction. For the optimum ATP concentration test, 0.25, 0.5, 1, 2, 3, and 4 mM ATP were used instead. For optimum salt concentration tests, 10, 50, 100, 150, 200, and 250 mM NaCl were used instead. For optimum metal type and concentration test, 1 and 5 mM MgCl_2_ or MnCl_2_ were used instead. The unwinding reactions were tracked using the SpectraMax M5E microplate reader (Molecular Devices, USA) by observing the increase in fluorescence signal (FAM mode) at 37°C for 20 min. Assays were performed in triplicate. Fluorescence signal change curves were generated using GraphPad Prism 9 (San Diego, USA). Error bars represent SD.

### DNA binding affinity analysis

Electrophoretic mobility shift assays (EMSA) were carried out to test the DNA binding affinity of SaDinG, according to reference (39). In total, 100 nM FAM-labeled 20 nt poly dT was mixed with different concentrations of SaDinG (0, 0.1, 0.2, 0.4, 0.8, 1.6, and 3.2 µM) in a 10 µL reaction volume containing 50 mM TRIS-HCl (pH 8.0), 80 mM NaCl, 1 mM TCEP, and 3.2% (vol/vol) glycerol. To examine the effects of ATP on DNA binding, we included different ATP concentrations (0, 0.625, 1.25, 2.5, 5, and 10 mM) in the reaction system. After incubation at 25°C for 10 min, the samples were separated on 5% native polyacrylamide gels in 0.5 × Tris-borate buffer. Gels were imaged in fluorescence mode (FAM) using ChemiScope6100 (Clinx Science Instruments, Shanghai). The binding fractions were calculated using Image J from three repeats, and the Kd values were calculated using GraphPad Prism 9 (San Diego, USA).

## RESULTS

### Domain arrangement and overall structure of SaDinG-ssDNA

**Fig 1:**
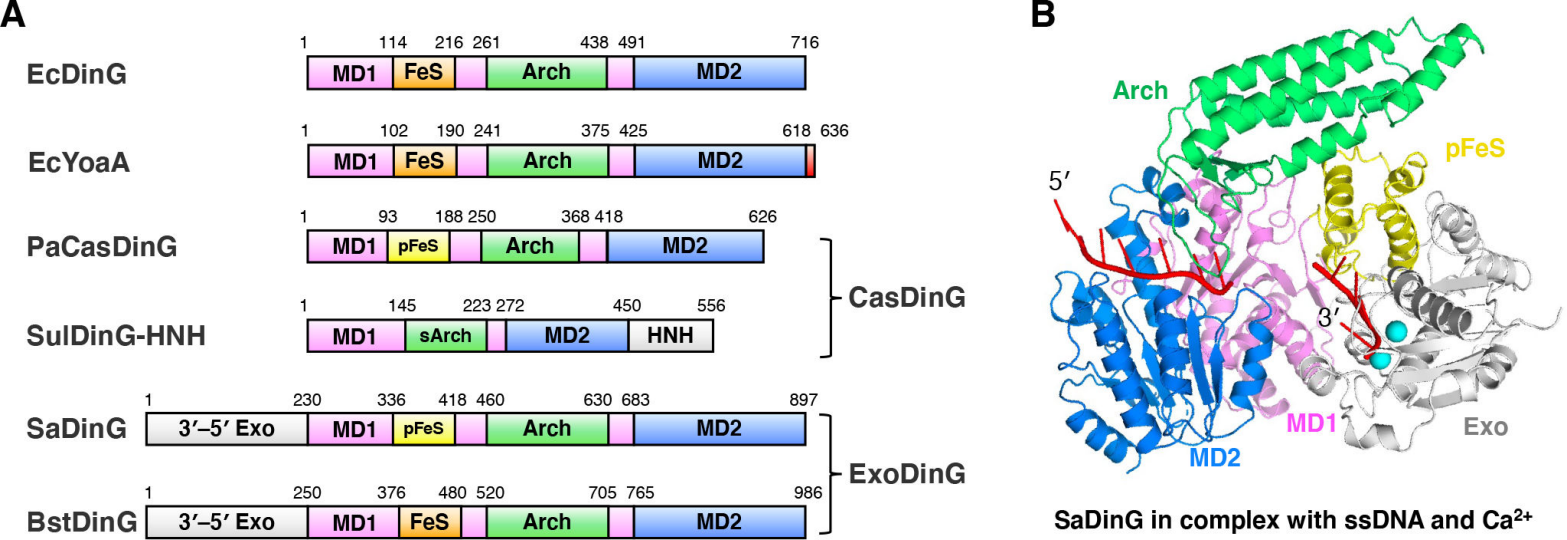
Domain arrangement and overall structure of SaDinG. (**A**) Schematic of the domain arrangements of EcDinG, EcYoaA, PaCasDinG, SulDinG-HNH, SaDinG, and BstDinG. MD1, motor domain 1; MD2, motor domain 2; FeS, the FeS cluster containing domain; pFeS, pseudo FeS domain; Arch, the Arch domain; sArch, the short version Arch domain; 3′–5′ Exo, the 3′–5′ exonuclease domain. (**B**) The overall structure of SaDinG-ssDNA-Ca^2+^. The 3′–5′ Exo, MD1, MD2, Arch, and pFeS domains were colored white, violet, marine, lime green, and yellow, respectively. ssDNA and Ca^2+^ were colored red and cyan, respectively.

The domain arrangements of SaDinG were analyzed and compared with four well-characterized bacterial DinG homologs: EcDinG, EcYoaA, PaCasDinG, and SulCasDinG-HNH (Fig. 1A). Among them, EcDinG and EcYoaA contain the four canonical domains found in XPD/DinG family members (Fig. 1A). Notably, PaCasDinG harbors a pseudo FeS (pFeS) domain that is unable to coordinate an FeS cluster due to the absence of cysteine residues, whereas SulCasDinG-HNH lacks any corresponding structural element in this region. Additionally, SulCasDinG-HNH features a shorter Arch domain and an extra C-terminal HNH nuclease domain (Fig. 1A). In certain bacterial lineages, including Bacillales, Lactobacillales, Ktedonobacterales, Caldilineales, Myxococcales, Fibrobacterales, and Tepidiformales, Bacteriovoracales, Eggerthellales, Coriobacteriales, Chloroflexales, Herpetosiphonales, Thermomicrobiales, Sphaerobacterales, Anaerolineales, Ardenticatenales, Thermoflexales, Phototrophicales, and Calditrichales, DinG has acquired an additional N-terminal 3′–5′ exonuclease (Exo) domain, defining a subgroup known as ExoDinG. Sequence analysis revealed that some ExoDinGs contain a pFeS domain instead of a canonical FeS domain (Fig. S1), with SaDinG serving as a representative example (Fig. 1A). Others, such as *Bacteriovorax stolpii* DinG (BstDinG), contain cysteine-rich clusters that may potentially coordinate an FeS cluster (Fig. 1A; Fig. S1).

The crystal structures of wild-type SaDinG in complex with ssDNA and Ca^2+^, as well as in complex with ssDNA alone, were determined at resolutions of 3.21 Å and 3.16 Å, respectively (Fig. 1B; Fig. S2). Molecular replacement was performed using the EcDinG-ssDNA complex structure (PDB: 6FWR) as a search model. The refined structures contain one protomer per asymmetric unit, with crystallographic statistics summarized in Table 1.

Although a 22-nt poly-d(T) ssDNA was used for co-crystallization, only 11 nucleotides were resolved in the SaDinG-ssDNA-Ca^2+^ complex structure, with seven bases interacting with the MD2 domain and four contacting the Exo domain. Two Ca^2+^ ions were identified at the nuclease active site of the SaDinG Exo domain (Fig. 1B). In contrast, when crystals were grown in a metal ion-free environment, no metal ions or DNA density were detected near the nuclease active site, and only four bases were observed interacting with the MD2 domain.

### Biochemical and structural characterization of the exonuclease activity of SaDinG

**Fig 2:**
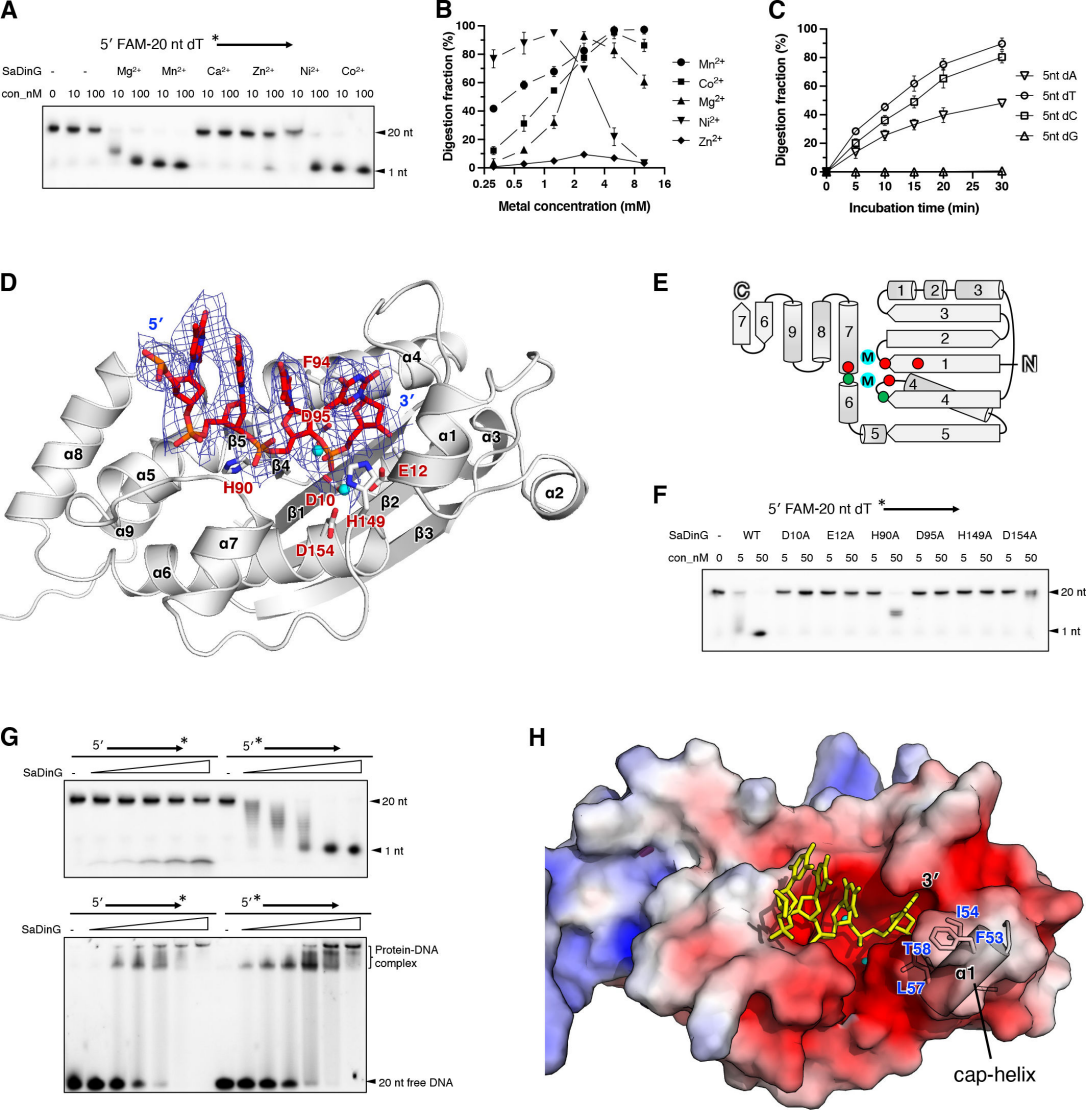
The exonuclease domain geometry and nuclease activity analysis of SaDinG. (**A**) Metal preference test. Hydrolysis of 100 nM 20 nt 5′ FAM labeled poly dT by SaDinG (10 or 100 nM) in the presence of indicated divalent metal cations (10 mM). (**B**) Divalent metal ion concentrations test. 100 nM 20 nt 5′ FAM labeled poly dT was incubated with 5 nM SaDinG (when MnCl_2_ or CoCl_2_ was used), or 10 nM SaDinG (when MgCl_2_, NiCl_2_, or ZnCl_2_). Different concentrations (0.313, 0.625, 1.25, 2.5, 5.0, and 10.0 mM) of divalent metal cations were introduced. Three technical replicates were conducted. (**C**) Optimum substrate bases determination; 0.8 mM substrates with 5 nt poly dA, dT, dC, or dG were incubated with 0.5 µM SaDinG and 10 mM Mn^2+^ for various durations (0, 5, 10, 15, 20, and 30 min), and the reaction products were resolved by TLC. Three technical replicates were conducted. (**D**) The zoomed-in view of the SaDinG exonuclease domain. The key residues involved in metal ion coordination and substrate interaction were shown as sticks and labeled. Ca^2+^ ions were represented as spheres and colored in cyan. Simulated annealing omit density (2Fo−Fc) contoured at 1.0 σ was shown in blue mesh around the DNA strand. (**E**) The topology diagram of the SaDinG exonuclease domain. The key residues involved in metal ion coordination and substrate interaction were shown as red dots and green dots, respectively. (**F**) Denaturing PAGE gel showing the reduced nuclease activity of point mutated SaDinG (alanine substitutions of key residues involved in metal ion coordination or substrates binding); 20 nt 5′ FAM labeled poly dT (100 nM) was incubated with different concentrations of SaDinG (5 and 50 nM) in the presence of 1 mM Mn^2+^. (**G**) Comparison of the digestion and binding activities towards 20 nt poly dT oligos labeled on different ends. The asterisk indicates the position of the fluorescent label. Upper, 100 nM DNA was incubated with varying concentrations of SaDinG (0, 1.25, 2.5, 5, 10, and 20 nM) in the presence of 1 mM Mn^2+^, and the resulting products were separated on a denaturing PAGE gel; bottom, 100 nM DNA was incubated with varying concentrations of SaDinG (0, 0.1, 0.2, 0.4, 0.8, 1.6, and 3.2 µM), and the resulting products were separated on a native-PAGE gel. (**H**) Analysis of the electrostatic properties of the SaDinG exonuclease domain. The electrostatic potential of this domain was determined using APBS, which was then projected onto the solvent-accessible surface of the structure at contouring levels of ±5 kT (depicted in blue/red). DNA bases were represented as sticks and colored yellow. The cap-helix (α1 of the SaDinG exonuclease domain) is illustrated as a cartoon, and the crucial residues on the cap-helix that inhibit the 3′ base progression are depicted as sticks.

To determine the optimal metal cofactors for the exonuclease activity of SaDinG, non-tagged SaDinG protein was purified and incubated with a 5′-FAM-labeled 20-nt ssDNA substrate in reaction buffers containing different metal cations. The results demonstrated that SaDinG efficiently hydrolyzes ssDNA down to mononucleotides, with the highest activity observed in the presence of Mn^2+^ or Co^2+^ as a cofactor when 10 mM metal ions were added to the reaction (Fig. 2A). Additionally, Ni^2+^ and Mg^2+^ also effectively supported exonuclease activity, whereas Zn^2+^ exhibited weak activity and Ca^2+^ exhibited no activity. The optimal concentrations for Mn^2+^, Co^2+^, Mg^2+^, Ni^2+^, and Zn^2+^ were determined to be 10.0, 5.0, 2.5, 1.25, and 2.5 mM, respectively (Fig. 2B).

To further investigate SaDinG’s substrate specificity, we compared its degradation efficiency toward 5-nt poly-dA, dT, dC, and dG using TLC analysis. The results revealed a digestion preference following the order dT >dC > dA >dG (Fig. 2C), indicating a strong affinity for pyrimidine-rich sequences. Additionally, SaDinG’s exonuclease activity was significantly affected by salt concentration, with the highest activity observed at lower salt concentrations (digestion efficiency followed the trend: 50 mM >100 mM > 150 mM >200 mM >250 mM >300 mM) (Fig. S3).

Structural analysis indicated that the Exo domain of SaDinG is composed of five β-strands arranged in the order of 32145 (↓↑↓↓↓), with three α-helices inserted between β3 and β4 and an additional α-helix inserted between β4 and β5. Following β5, a helical bundle composed of five α-helices is present. Additionally, a β-hairpin structural element (β6–β7) serves as a linker between the Exo domain and MD1 (Fig. 2D and E).

To identify structural homologs, the SaDinG Exo domain was analyzed using the Dali server. The top-ranked homologs were all members of the DEDDh-type 3′–5′ exonuclease family, including the N-terminal domain (NTD) of *E. coli* Exonuclease I (PDB: 4JS4; Z-score = 20.7, RMSD = 3.0 Å, identity = 18%) (40), *E. coli* RNase T (PDB code: 3V9X; Z-score = 19.5, RMSD = 2.6, identity = 23%) (41), *E. coli* Cap18 (PDB code: 7T2S; Z-score = 19.1, RMSD = 2.6, identity = 19%) (42), and the ε subunit of *E. coli* DNA polymerase III (DnaQ) (PDB code: 5M1S; Z-score = 18.2, RMSD = 2.9, identity = 26%) (Fig. S4) (43).

As a canonical DEDDh type 3′–5′ exonuclease, SaDinG features a conserved catalytic core consisting of five residues that coordinate the divalent metal ion and the scissile phosphate group: aspartate (D10) and glutamate (E12) at the C-terminal end of β1, aspartate (D95) at the N-terminal end of α4, and histidine (H149) and aspartate (D154) at the N-terminal end of α7 (Fig. 2D and E). These DEDD residues coordinate two metal ions at the active site, with H149 specifically interacting with the scissile phosphate group and activating the nucleophilic water molecule. Additionally, a conserved histidine (H90) at the C-terminal end of β4 interacts with the phosphate group of the second nucleotide (Fig. 2D). Alanine substitutions of these residues nearly abolished exonuclease activity, with the exception of the H90A mutant. Although the H90A variant retained partial activity, it generated a larger intermediate product (Fig. 2F). Extended incubation experiments demonstrated that the H90A mutant could not completely digest ssDNA into 1-nt fragments (Fig. S5), indicating H90’s critical role in threading ssDNA into the nuclease active site.

To further characterize its enzymatic properties, we compared the digestion efficiencies of SaDinG on 20-nucleotide poly-dT substrates labeled with FAM at either the 3′ or 5′ end. Notably, SaDinG exhibited significantly higher activity on the 5′-labeled substrate, despite comparable binding affinities (Fig. 2G). This enzymatic preference resembles that of another DEDDh-type 3′–5′ exonuclease, *B. subtilis* MrfB, where a fluorophore at the 3′ end inhibits digestion (44). Structural analysis revealed a conserved hydrophobic cap-helix near the nuclease active site, forming a pocket that likely accommodates the 3′-terminal base during digestion (Fig. 2H; Fig. S4). This structural feature appears to be crucial for recognizing the 3′ end of the DNA substrate and ensuring 3′–5′ exonuclease activity. Similar cap-helices have been identified in other DEDDh-type 3′–5′ exonucleases, including *E. coli* Exonuclease I (PDB: 4JS4), *E. coli* RNase T (PDB: 3V9X), *E. coli* Cap18 (PDB: 7T2S), *E. coli* DnaQ (PDB: 5M1S), and *B. subtilis* MrfB (PDB: 8UN9) (Fig. S4).

### Biochemical and structural characterization of the helicase activity of SaDinG

**Fig 3:**
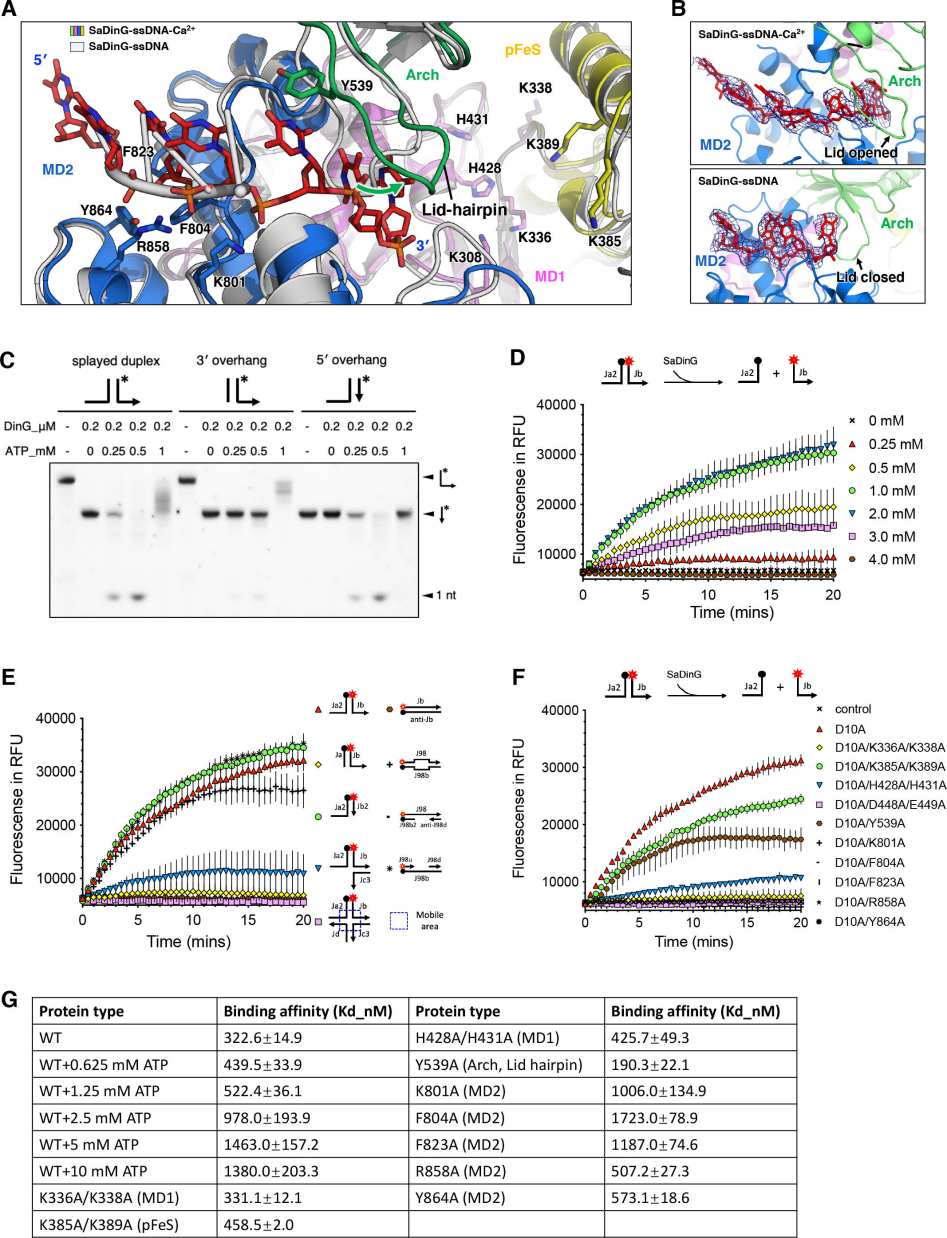
The helicase activity analysis of SaDinG. (**A**) Zoomed-in view of the DNA binding channel in the helicase domain of SaDinG. The (potential) key residues involved in DNA binding were shown as sticks and labeled. The SaDinG-ssDNA-Ca^2+^ complex was colored by domains, consistent with Fig. 1, whereas the SaDinG-ssDNA structure was shown in white gray. The green arrow shows the opening direction of the lid-hairpin upon SaDinG binding to the long DNA strand. (**B**) The electronic density of DNA in the helicase domain’s binding channel. Upper, data from SaDinG-ssDNA-Ca^2+^ complex; bottom, data from SaDinG-ssDNA complex. Simulated annealing omit density (2Fo−Fc) contoured at 1.0 σ was shown in blue mesh around the DNA strand. (**C**) Digestion test on substrates with different overhangs. 100 nM 5′ FAM labeled splayed duplex, 3′ overhang, or 5′ overhang substrate was incubated with 0.2 µM SaDinG and 5 mM MgCl_2_. Different concentrations (0, 0.25, 0.5, and 1 mM) of ATP were introduced, and the resulting products were separated on a TBE-urea denaturing PAGE gel. The starting point of the arrow represents the 5' end of the DNA, the endpoint represents the 3′ end, and the asterisk indicates the position of the fluorescent label. (**D**) Real-time unwinding assays to test the optimum ATP concentration. The real-time unwound fractions of splayed duplex were monitored at different concentrations (0, 0.25, 0.5, 1.0, 2.0, 3.0, and 4.0 mM) of ATP and shown as curves; 25 nM substrate was incubated with 0.5 µM nuclease dead SaDinG (D10A) and 1 mM MgCl_2_, and specific concentration of ATP. Each assay was conducted in triplicate. Error bars represent SD. (**E**) Real-time unwinding assays on different substrates. The real-time unwound fractions of different substrates (splayed duplex, annealed by Jb and Ja2; 3′-overhang, annealed by Jb and Ja; 5′-overhang, annealed by Jb2 and Ja2; double-flap, annealed by Jb, Ja2, and Jc3; holiday junction, annealed by Jb, Ja2, Jc3, and Jd; duplex, annealed by Jb and anti-Jb; bubble, annealed by J98 and J98b; gapped duplex 1, annealed by J98, J98b2, and anti-J98d; gapped duplex 2, annealed by J98u, J98d, and J98b) were monitored and shown as curves. 25 nM substrate was incubated with 0.5 µM nuclease dead SaDinG (D10A), 1.0 mM ATP, and 1 mM MgCl_2_. Each assay was conducted in triplicate. Error bars represent SD. (**F**) Real-time unwinding assays of SaDinG mutants toward splayed duplex. The reaction conditions were the same as (**E**). (**G**) DNA binding affinity of SaDinG mutants. The dissociation constants (Kd values) for ssDNA binding affinity were determined from binding fraction curves obtained by EMSA assays. The corresponding residues are annotated with parentheses indicating their located structural domains and elements. Various ATP concentrations were added to evaluate inhibitory effects. Each assay was conducted in triplicate.

Protein-ssDNA interaction analysis revealed that the DNA binding mode of the MD2 domain in SaDinG closely resembles that of EcDinG and PaCasDinG (Fig. 3A; Fig. S6). In the SaDinG-ssDNA-Ca^2+^ complex structure, a total of seven nucleotide bases are contacted by the MD2 domain (Fig. 3B). Several conserved aromatic residues (F804, F823, and Y864) participate in base/ribose ring stacking interactions, whereas basic residues (K801 and R858) engage in polar hydrogen bonding interactions (Fig. 3A; Fig. S1 and S6). Although no DNA density was observed in the MD1 and pFeS domains, a series of positively charged residues (K336, K338, K385, K389, H428, and H431) line the channel, suggesting a potential role in DNA binding (Fig. 3A). Additionally, a lid hairpin structure within the Arch domain of SaDinG was identified, enclosing the DNA chain. In the absence of DNA binding, this lid hairpin adopts a "closed" conformation, introducing steric hindrance that prevents DNA association (Fig. 3A and B). Structural comparison revealed that PaCasDinG also possesses a similar lid hairpin structure, which encloses the DNA chain (PDB code: 7XF1) (Fig. S6). In its DNA-free state, this lid hairpin interacts with the pFeS domain, narrowing the DNA binding channel in PaCasDinG (PDB code: 7XEX). In contrast, EcDinG (PDB code: 6FWR) lacks a typical lid hairpin (Fig. S6).

Previously, SaDinG was reported to lack helicase activity (27). To validate this conclusion, we examined its nuclease activity against splayed duplex, 3′ overhang, and 5′ overhang substrates in the presence of varying ATP concentrations. In the absence of ATP, SaDinG digested only the ssDNA portion of these substrates (Fig. 3C). Notably, at low ATP concentrations (0.25–0.5 mM), SaDinG demonstrated the ability to degrade duplex regions in both splayed duplex and 5′-overhang substrates, producing fully digested 1-nt products. These observations indicate that SaDinG possesses helicase activity with a preference for 5′-overhang ssDNA-contained substrate. Interestingly, elevated ATP concentration (1 mM) substantially reduced digestion efficiency (Fig. 3C). Similarly, elevated ATP inhibited the SaDinG nuclease activity on ssDNA substrates (Fig. S7), with significant inhibition becoming evident at concentrations between 0.625 and 1.25 mM—consistent with the results shown in Fig. 3C. In contrast, dATP had no obvious inhibitory effect on nuclease activity (Fig. S7A). Increasing Mg^2+^ concentration counteracted ATP-mediated inhibition (Fig. S7B), suggesting that the inhibition effect is likely due to ATP-dependent metal chelation.

To determine the optimal ATP concentration for SaDinG helicase activity, we performed real-time unwinding assays (Fig. 3D). A 25 nM splayed duplex substrate, labeled with a 5′ fluorophore (FAM) and a 3′ quencher (Dabsyl) on each strand, was incubated with 0.5 µM nuclease-dead SaDinG (D10A), and fluorescence intensity was monitored to quantify strand separation. The results showed that the optimal ATP concentration for helicase activity was 1.0–2.0 mM. Consistent with the nuclease assay, helicase activity was inhibited at higher ATP concentrations. Substrate specificity analysis demonstrated that SaDinG preferentially unwound splayed duplex and 5′-overhang substrate while showing minimal activity on the 3′-overhang substrate (Fig. 3E), consistent with the digestion assay results. Furthermore, SaDinG exhibited 5′–3′ helicase activity on various DNA architectures including double-flap, bubble, and gapped duplex substrates (Fig. 3E), suggesting its multifunctional role in DNA metabolic processes *in vivo*. The optimal NaCl concentration for unwinding was 50 mM, mirroring the nuclease activity conditions (Fig. S8). Moreover, Mg^2+^ was found to be a more effective cofactor than Mn^2+^. In contrast to its exonuclease activity, SaDinG’s helicase function showed greater efficiency at lower metal ion concentrations (1 mM) compared with higher concentrations (5 mM), with similar effects observed for both Mg^2+^ and Mn^2+^ (Fig. S8). This suggests minimal influence of metal chelation on helicase activity.

To further investigate key residues involved in DNA binding and translocation, we introduced alanine substitutions in selected residues on the nuclease-dead background (D10A). Real-time unwinding assays demonstrated that K336A/K338A, K801A, F804A, F823A, R858A, and Y864A mutations resulted in a complete loss of helicase activity, whereas K385A/K389A, H428A/H431A, and Y539A mutations caused partial activity reduction (Fig. 3F). EMSA was performed to assess DNA binding affinities, with dissociation constants (Kd values) derived from binding fraction curves across three replicates. K801A, F804A, and F823A mutants exhibited significantly reduced DNA binding affinities (Kd values increased more than 2-fold), whereas K336A/K338A, K385A/K389A, H428A/H431A, R858A, and Y864A mutants showed moderate affinity reductions (Fig. 3G). Interestingly, the lid hairpin mutant (Y539A) displayed an increased DNA binding affinity (Kd value halved relative to wild-type) but exhibited weaker helicase activity (Fig. 3G). Notably, in real-time unwinding assays, the Y539A mutant not only showed reduced unwinding efficiency but also exhibited partial DNA re-annealing (Fig. 3F), suggesting a defect in unidirectional translocation. These findings imply that the lid hairpin functions as a molecular ratchet to ensure unidirectional DNA translocation. Furthermore, ATP addition increased the DNA binding Kd value (Fig. 3G), providing a potential explanation for the observed inhibition of SaDinG helicase activity at elevated ATP concentrations. In addition to metal chelation, this reduced DNA binding affinity may also contribute to ATP-mediated inhibition of SaDinG’s exonuclease activity.

### *S. aureus* DinG plays a role in repairing DNA damage induced by MMC

**Fig 4:**
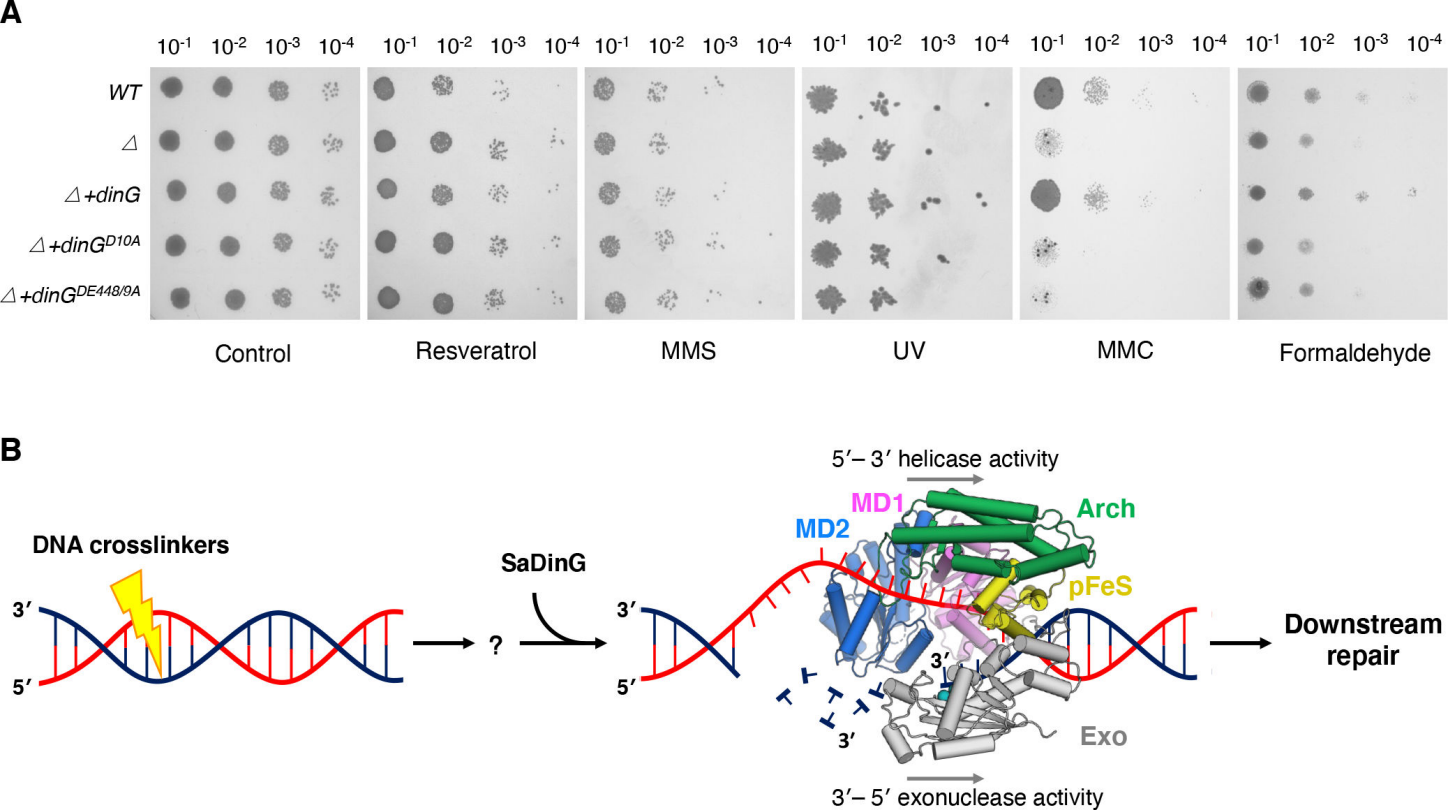
The biological role of SaDinG in DNA crosslink repair. (**A**) The phenotypes of deletion mutant and complementation strains. The WT, SaDinG deleted strain (*△*), SaDinG deleted strains complemented with *dinG*, *dinG^D10A^*, or *dinG^DE448/9A^* (*△+dinG, △+dinGD^D10A^,* or *△+dinGDE^DE448/9A^*) were treated with 100 µg/mL of resveratrol (supplemented in the agar plate), 10 mM of MMS (15 mins), UV at a dose of 100 J/m^2^, and 8 µg/mL of MMC (supplemented in the agar plate). Cells were diluted and dotted on plates. Plates were cultured for 2–3 days at 30°C. (**B**) Model for SaDinG participates in DNA crosslink repair. SaDinG initiates repair by unwinding crosslinked DNA (5′−3′ helicase activity) while its exonuclease concurrently excises the damaged strand (3′−5′), creating a repair intermediate for downstream factors.

Previous studies reported that SaDinG is not regulated by the SOS response repressor LexA (25). Given its additional exonuclease activity, it remains unclear whether SaDinGs have acquired other biological functions. To address this, we deleted the *dinG* gene in the *S. aureus* RN4220 strain using a pKOR1-mediated allelic replacement method and conducted multiple phenotype assays. The SaDinG deletion mutant exhibited no effects on cell growth and resistance to resveratrol (an SOS response inducer [33]), UV, and MMS resistance (Fig. 4A). However, the mutant displayed increased sensitivity to MMC or formaldehyde treatment (Fig. 4A). This phenotype can be restored by complementing with the wild-type SaDinG but not with nuclease-dead (*dinG^D10A^*) or ATPase-dead mutants (*dinG^D448A/E449A^*).

Given that both MMC and formaldehyde would induce DNA intrastrand and interstrand crosslinks, these results demonstrate that SaDinG might be essential for crosslink repair in *S. aureus*, dependent on both its nuclease and ATPase activities. Based on these findings, we proposed a mechanistic model wherein SaDinG operates as an integrated nuclease-helicase machine specialized for DNA crosslink repair (Fig. 4B). The repair process initiates when SaDinG’s helicase domain unwinds the crosslinked DNA duplex while translocating processively in the 5′–3′ direction along one strand. Concurrently, the exonuclease domain excises the opposing damaged strand in the 3′–5′ direction, precisely removing the lesion-containing segment. This coordinated bidirectional processing generates a repair intermediate that is subsequently processed by downstream repair factors. However, key aspects of this model, including SaDinG’s lesion recognition mechanism and potential requirement for accessory proteins, require further experimental validation.

## DISCUSSION

Previous studies of *E. coli* DinG and archaeal XPD have demonstrated that disruption of the FeS-binding motif results in loss of helicase activity (5, 23, 24). Interestingly, despite the natural absence of an FeS cluster, SaDinG retains robust helicase activity. Our mutational analysis suggests that two basic residues (K385 and K389) within the pFeS domain contribute to this activity. Notably, PaCasDinG, which also lacks an FeS cluster, exhibits strong helicase activity. Structural analyses have shown that the pFeS domain is essential for PaCasDinG helicase function, undergoing significant conformational rearrangements upon ssDNA binding. Point mutations in key DNA-binding residues (G95A/R96A/P97A) within this domain severely impair helicase activity (14). These findings suggest that although some DinG homologs may have lost the capacity for redox sensing and regulation, the structural elements of the pFeS domain remain integral to DNA binding and enzymatic function.

Additionally, we identified a lid-hairpin structural element in the Arch domain of SaDinG, similar to that observed in PaCasDinG. Our biochemical data suggest that this lid-hairpin functions as a molecular ratchet, likely preventing substrate backtracking during DNA translocation. This structural feature may serve as a key determinant in ensuring processive helicase activity.

ATP-dependent inhibition of nuclease activity has been reported in multiple nucleases, including anti-phage defense proteins such as GajAB, PtuAB, and Sir2-HerA, where small molecules like ATP occupy active sites or DNA-binding channels to modulate enzymatic activity (45–47). In this study, we confirmed that the inhibition of nuclease activity by elevated ATP results from both ATP-dependent metal chelation and ATP-mediated reductions in substrate affinity, whereas helicase inhibition is primarily driven by the ATP-mediated decrease in substrate affinity. This observation may also explain why McRobbie et al. previously failed to detect significant helicase activity in SaDinG (27). In *E. coli*, intracellular ATP concentrations exceed 3 mM (48). The intracellular ATP concentration in *S. aureus* under physiological conditions remains unknown. If it is similar to that in *E. coli*, this would suggest that SaDinG is probably inactive under physiological conditions in cells. In some anti-phage defense systems, it has been proposed that phage infection induces extensive transcription and metabolic activity, depleting cellular ATP levels and thereby activating these defense proteins. Although it is uncertain whether SaDinG is part of the anti-phage defense system, there may be a mechanism regulating its activity through modulation of cellular ATP concentration.

MMC, originally isolated from *Streptomyces lavendulae* (49), exhibits both antibiotic and anti-tumor properties. MMC intercalates into DNA, preferentially binding to guanine residues, where it forms crosslinks that inhibit DNA replication (50). Similar to SaDinG, the deletion of *mrfA* and *mrfB* results in exclusive sensitivity to MMC in *B. subtilis*. MrfB is a DEDDh family 3′–5′ exonuclease, and MrfA is a SF2 family 3′–5′ DNA helicase (44, 51). They cooperatively play roles in removing the monoadduct or the intra-strand crosslink caused by MMC (52). From a structural and biochemical perspective, SaDinG appears to be a natural fusion of MrfA and MrfB, combining both 3′–5′ exonuclease and helicase activities within a single polypeptide. However, a key distinction lies in their DNA unwinding polarity—SaDinG unwinds DNA with a 5′–3′ polarity, whereas MrfA operates in the opposite direction. Whether SaDinG and the MrfAB complex share mechanistic similarities in resolving DNA lesions remains an open question. The presence of SaDinG in certain bacteria may reflect an evolutionary adaptation to environments rich in MMC-producing microbes, akin to the proposed role of MrfAB. Notably, SaDinG also contributes to resistance against formaldehyde, another crosslinking agent, underscoring its broader role in DNA crosslink repair. The integration of exonuclease and helicase domains within SaDinG may provide functional advantages over other DinG homologs, such as enhanced efficiency in processing crosslinked DNA. Key unresolved questions include whether SaDinG exhibits sequence or base preferences during substrate recognition and hydrolysis, and how such specificity influences its response to DNA damage. Further structural and mechanistic studies will be essential to unravel the molecular basis of SaDinG’s physiological functions and its potential unique role in genome maintenance.

## Data Availability

All data generated or analyzed during this study are included in this published article, its supplementary information files and publicly available repositories. Atomic coordinates and structure factors for the reported crystal structures have been deposited with the Protein Data Bank (http://www.wwpdb.org) under accession number 8ZEF (DOI: 10.2210/pdb8zef/pdb) and 9II8 (DOI: 10.2210/pdb9ii8/pdb).

## SUPPLEMENTAL MATERIAL

Supplemental material mbio.00884-25-s0001.pdf Table S1 and Fig. S1 to Fig. S8.

ASM does not own the copyrights to Supplemental Material that may be linked to, or accessed through, an article. The authors have granted ASM a non-exclusive, world-wide license to publish the Supplemental Material files. Please contact the corresponding author directly for reuse.

## References

[1] White MF. 2009. Structure, function and evolution of the XPD family of iron-sulfur-containing 5'-->3' DNA helicases. Biochem Soc Trans 37:547–551. doi:10.1042/BST0370547 19442249

[2] Cheng K. 2025. Structure, function and evolution of the bacterial DinG-like proteins. Comput Struct Biotechnol J 27:1124–1139. doi:10.1016/j.csbj.2025.03.023 40206346 PMC11981726

[3] Lewis LK, Jenkins ME, Mount DW. 1992. Isolation of DNA damage-inducible promoters in Escherichia coli: regulation of polB (dinA), dinG, and dinH by LexA repressor. J Bacteriol 174:3377–3385. doi:10.1128/jb.174.10.3377-3385.1992 1577702 PMC206008

[4] Voloshin ON, Vanevski F, Khil PP, Camerini-Otero RD. 2003. Characterization of the DNA damage-inducible helicase DinG from Escherichia coli. J Biol Chem 278:28284–28293. doi:10.1074/jbc.M301188200 12748189

[5] Ren B, Duan X, Ding H. 2009. Redox control of the DNA damage-inducible protein DinG helicase activity via its iron-sulfur cluster. J Biol Chem 284:4829–4835. doi:10.1074/jbc.M807943200 19074432 PMC2643519

[6] Voloshin ON, Camerini-Otero RD. 2007. The DinG protein from Escherichia coli is a structure-specific helicase. J Biol Chem 282:18437–18447. doi:10.1074/jbc.M700376200 17416902

[7] Bharti SK, Sommers JA, George F, Kuper J, Hamon F, Shin-ya K, Teulade-Fichou MP, Kisker C, Brosh RM Jr. 2013. Specialization among iron-sulfur cluster helicases to resolve G-quadruplex DNA structures that threaten genomic stability. J Biol Chem 288:28217–28229. doi:10.1074/jbc.M113.496463 23935105 PMC3784731

[8] Cheng Z, Caillet A, Ren B, Ding H. 2012. Stimulation of Escherichia coli DNA damage inducible DNA helicase DinG by the single-stranded DNA binding protein SSB. FEBS Lett 586:3825–3830. doi:10.1016/j.febslet.2012.09.032 23036643 PMC3483465

[9] Frye SA, Beyene GT, Namouchi A, Gómez-Muñoz M, Homberset H, Kalayou S, Riaz T, Tønjum T, Balasingham SV. 2017. The helicase DinG responds to stress due to DNA double strand breaks. PLoS One 12:e0187900. doi:10.1371/journal.pone.0187900 29121674 PMC5679670

[10] Courcelle J, Khodursky A, Peter B, Brown PO, Hanawalt PC. 2001. Comparative gene expression profiles following UV exposure in wild-type and SOS-deficient Escherichia coli. Genetics 158:41–64. doi:10.1093/genetics/158.1.41 11333217 PMC1461638

[11] Sutera VA, Sass TH, Leonard SE, Wu L, Glass DJ, Giordano GG, Zur Y, Lovett ST. 2021. Genetic analysis of DinG family helicase YoaA and its interaction with replication clamp loader protein HolC in Escherichia coli. J Bacteriol 203:e0022821. doi:10.1128/JB.00228-21 34181484 PMC8378479

[12] Weeks-Pollenz SJ, Ali Y, Morris LA, Sutera VA, Dudenhausen EE, Hibnick M, Lovett ST, Bloom LB. 2023. Characterization of the Escherichia coli XPD/Rad3 iron-sulfur helicase YoaA in complex with the DNA polymerase III clamp loader subunit chi (χ). J Biol Chem 299:102786. doi:10.1016/j.jbc.2022.102786 36509145 PMC9826845

[13] Crowley VM, Catching A, Taylor HN, Borges AL, Metcalf J, Bondy-Denomy J, Jackson RN. 2019. A type IV-A CRISPR-Cas system in Pseudomonas aeruginosa mediates RNA-guided plasmid interference in vivo. CRISPR J 2:434–440. doi:10.1089/crispr.2019.0048 31809194 PMC6919247

[14] Cui N, Zhang JT, Liu Y, Liu Y, Liu XY, Wang C, Huang H, Jia N. 2023. Type IV-A CRISPR-Csf complex: assembly, dsDNA targeting, and CasDinG recruitment. Mol Cell 83:2493–2508. doi:10.1016/j.molcel.2023.05.036 37343553

[15] Domgaard H, Cahoon C, Armbrust MJ, Redman O, Jolley A, Thomas A, Jackson RN. 2023. CasDinG is a 5’-3’ dsDNA and RNA/DNA helicase with three accessory domains essential for type IV CRISPR immunity. Nucleic Acids Res 51:8115–8132. doi:10.1093/nar/gkad546 37395408 PMC10450177

[16] Guo X, Sanchez-Londono M, Gomes-Filho JV, Hernandez-Tamayo R, Rust S, Immelmann LM, Schäfer P, Wiegel J, Graumann PL, Randau L. 2022. Characterization of the self-targeting Type IV CRISPR interference system in Pseudomonas oleovorans. Nat Microbiol 7:1870–1878. doi:10.1038/s41564-022-01229-2 36175516

[17] Altae-Tran H, Kannan S, Suberski AJ, Mears KS, Demircioglu FE, Moeller L, Kocalar S, Oshiro R, Makarova KS, Macrae RK, Koonin EV, Zhang F. 2023. Uncovering the functional diversity of rare CRISPR-Cas systems with deep terascale clustering. Science 382:eadi1910. doi:10.1126/science.adi1910 37995242 PMC10910872

[18] Liu H, Rudolf J, Johnson KA, McMahon SA, Oke M, Carter L, McRobbie AM, Brown SE, Naismith JH, White MF. 2008. Structure of the DNA repair helicase XPD. Cell 133:801–812. doi:10.1016/j.cell.2008.04.029 18510925 PMC3326533

[19] Wolski SC, Kuper J, Hänzelmann P, Truglio JJ, Croteau DL, Van Houten B, Kisker C. 2008. Crystal structure of the FeS cluster-containing nucleotide excision repair helicase XPD. PLoS Biol 6:e149. doi:10.1371/journal.pbio.0060149 18578568 PMC2435149

[20] Kuper J, Wolski SC, Michels G, Kisker C. 2012. Functional and structural studies of the nucleotide excision repair helicase XPD suggest a polarity for DNA translocation. EMBO J 31:494–502. doi:10.1038/emboj.2011.374 22081108 PMC3261550

[21] Constantinescu-Aruxandei D, Petrovic-Stojanovska B, Penedo JC, White MF, Naismith JH. 2016. Mechanism of DNA loading by the DNA repair helicase XPD. Nucleic Acids Res 44:2806–2815. doi:10.1093/nar/gkw102 26896802 PMC4824113

[22] Fan L, Fuss JO, Cheng QJ, Arvai AS, Hammel M, Roberts VA, Cooper PK, Tainer JA. 2008. XPD helicase structures and activities: insights into the cancer and aging phenotypes from XPD mutations. Cell 133:789–800. doi:10.1016/j.cell.2008.04.030 18510924 PMC3055247

[23] Cheng K, Wigley DB. 2018. DNA translocation mechanism of an XPD family helicase. Elife 7:e42400. doi:10.7554/eLife.42400 30520735 PMC6300356

[24] Peissert S, Sauer F, Grabarczyk DB, Braun C, Sander G, Poterszman A, Egly JM, Kuper J, Kisker C. 2020. In TFIIH the Arch domain of XPD is mechanistically essential for transcription and DNA repair. Nat Commun 11:1667. doi:10.1038/s41467-020-15241-9 32245994 PMC7125077

[25] Cirz RT, Jones MB, Gingles NA, Minogue TD, Jarrahi B, Peterson SN, Romesberg FE. 2007. Complete and SOS-mediated response of Staphylococcus aureus to the antibiotic ciprofloxacin. J Bacteriol 189:531–539. doi:10.1128/JB.01464-06 17085555 PMC1797410

[26] Au N, Kuester-Schoeck E, Mandava V, Bothwell LE, Canny SP, Chachu K, Colavito SA, Fuller SN, Groban ES, Hensley LA, O’Brien TC, Shah A, Tierney JT, Tomm LL, O’Gara TM, Goranov AI, Grossman AD, Lovett CM. 2005. Genetic composition of the Bacillus subtilis SOS system. J Bacteriol 187:7655–7666. doi:10.1128/JB.187.22.7655-7666.2005 16267290 PMC1280312

[27] McRobbie AM, Meyer B, Rouillon C, Petrovic-Stojanovska B, Liu H, White MF. 2012. Staphylococcus aureus DinG, a helicase that has evolved into a nuclease. Biochem J 442:77–84. doi:10.1042/BJ20111903 22166102 PMC3270479

[28] Cheng K, Xu H, Chen X, Wang L, Tian B, Zhao Y, Hua Y. 2016. Structural basis for DNA 5´-end resection by RecJ. Elife 5:e14294. doi:10.7554/eLife.14294 27058167 PMC4846377

[29] Bae T, Schneewind O. 2006. Allelic replacement in Staphylococcus aureus with inducible counter-selection. Plasmid 55:58–63. doi:10.1016/j.plasmid.2005.05.005 16051359

[30] Chen Y, Ji S, Sun L, Wang H, Zhu F, Chen M, Zhuang H, Wang Z, Jiang S, Yu Y, Chen Y. 2022. The novel fosfomycin resistance gene fosY is present on a genomic island in CC1 methicillin-resistant Staphylococcus aureus. Emerg Microbes Infect 11:1166–1173. doi:10.1080/22221751.2022.2058421 35332834 PMC9037201

[31] Cheng K, Chen X, Xu G, Wang L, Xu H, Yang S, Zhao Y, Hua Y. 2015. Biochemical and functional characterization of the NurA-HerA complex from Deinococcus radiodurans. J Bacteriol 197:2048–2061. doi:10.1128/JB.00018-15 25868646 PMC4438212

[32] Cheng K, Xu Y, Chen X, Lu H, He Y, Wang L, Hua Y. 2020. Participation of RecJ in the base excision repair pathway of Deinococcus radiodurans. Nucleic Acids Res 48:9859–9871. doi:10.1093/nar/gkaa714 32870272 PMC7515722

[33] Liu L, Ingmer H, Vestergaard M. 2021. Genome-wide identification of resveratrol intrinsic resistance determinants in Staphylococcus aureus. Antibiotics (Basel) 10:82. doi:10.3390/antibiotics10010082 33467002 PMC7829806

[34] Wang Y, Hao W, Guo Z, Sun Y, Wu Y, Sun Y, Gao T, Luo Y, Jin L, Yang J, Cheng K. 2024. Structural and functional investigation of the DHH/DHHA1 family proteins in Deinococcus radiodurans. Nucleic Acids Res 52:7142–7157. doi:10.1093/nar/gkae451 38804263 PMC11229311

[35] Liu K, Zhou H, Xu Q, Kong H-T, Zhang K-H, Wang W-W, Li M-J, Wang Z-J, Pan Q-Y, Wang X-Y, Yu F, Wang Q-S. 2023. BL02U1: the relocated macromolecular crystallography beamline at the Shanghai Synchrotron Radiation Facility. Nucl Sci Tech 34:193. doi:10.1007/s41365-023-01348-3

[36] Yu F, Wang Q, Li M, Zhou H, Liu K, Zhang K, Wang Z, Xu Q, Xu C, Pan Q, He J. 2019. Aquarium: an automatic data-processing and experiment information management system for biological macromolecular crystallography beamlines. J Appl Crystallogr 52:472–477. doi:10.1107/S1600576719001183

[37] Adams PD, Afonine PV, Bunkóczi G, Chen VB, Davis IW, Echols N, Headd JJ, Hung L-W, Kapral GJ, Grosse-Kunstleve RW, McCoy AJ, Moriarty NW, Oeffner R, Read RJ, Richardson DC, Richardson JS, Terwilliger TC, Zwart PH. 2010. PHENIX: a comprehensive Python-based system for macromolecular structure solution. Acta Crystallogr D Biol Crystallogr 66:213–221. doi:10.1107/S0907444909052925 20124702 PMC2815670

[38] Emsley P, Lohkamp B, Scott WG, Cowtan K. 2010. Features and development of Coot. Acta Crystallogr D Biol Crystallogr 66:486–501. doi:10.1107/S0907444910007493 20383002 PMC2852313

[39] Sun Y, Yang J, Xu G, Cheng K. 2022. Biochemical and structural study of RuvC and YqgF from Deinococcus radiodurans. mBio 13:e0183422. doi:10.1128/mbio.01834-22 36000732 PMC9601230

[40] Korada SKC, Johns TD, Smith CE, Jones ND, McCabe KA, Bell CE. 2013. Crystal structures of Escherichia coli exonuclease I in complex with single-stranded DNA provide insights into the mechanism of processive digestion. Nucleic Acids Res 41:5887–5897. doi:10.1093/nar/gkt278 23609540 PMC3675492

[41] Hsiao YY, Duh Y, Chen YP, Wang YT, Yuan HS. 2012. How an exonuclease decides where to stop in trimming of nucleic acids: crystal structures of RNase T-product complexes. Nucleic Acids Res 40:8144–8154. doi:10.1093/nar/gks548 22718982 PMC3439924

[42] Liang Q, Richey ST, Ur SN, Ye Q, Lau RK, Corbett KD. 2022. Structure and activity of a bacterial defense-associated 3′-5′ exonuclease. Protein Sci 31:e4374. doi:10.1002/pro.4374 35762727 PMC9214754

[43] Fernandez-Leiro R, Conrad J, Yang J-C, Freund SMV, Scheres SHW, Lamers MH. 2017. Self-correcting mismatches during high-fidelity DNA replication. Nat Struct Mol Biol 24:140–143. doi:10.1038/nsmb.3348 28067916 PMC5300136

[44] Manthei KA, Munson LM, Nandakumar J, Simmons LA. 2024. Structural and biochemical characterization of the mitomycin C repair exonuclease MrfB. Nucleic Acids Res 52:6347–6359. doi:10.1093/nar/gkae308 38661211 PMC11194089

[45] Cheng R, Huang F, Lu X, Yan Y, Yu B, Wang X, Zhu B. 2023. Prokaryotic Gabija complex senses and executes nucleotide depletion and DNA cleavage for antiviral defense. Cell Host Microbe 31:1331–1344. doi:10.1016/j.chom.2023.06.014 37480847

[46] Li Y, Shen Z, Zhang M, Yang X-Y, Cleary SP, Xie J, Marathe IA, Kostelic M, Greenwald J, Rish AD, Wysocki VH, Chen C, Chen Q, Fu T-M, Yu Y. 2024. PtuA and PtuB assemble into an inflammasome-like oligomer for anti-phage defense. Nat Struct Mol Biol 31:413–423. doi:10.1038/s41594-023-01172-8 38177683 PMC12318543

[47] Tang D, Chen Y, Chen H, Jia T, Chen Q, Yu Y. 2023. Multiple enzymatic activities of a Sir2-HerA system cooperate for anti-phage defense. Mol Cell 83:4600–4613. doi:10.1016/j.molcel.2023.11.010 38096825

[48] Buckstein MH, He J, Rubin H. 2008. Characterization of nucleotide pools as a function of physiological state in Escherichia coli. J Bacteriol 190:718–726. doi:10.1128/JB.01020-07 17965154 PMC2223692

[49] Hata T, Sugawara R. 1956. Mitomycin, a new antibiotic from Streptomyces. II. Description of the strain. J Antibiot (Tokyo) 9:147–151. 13385187

[50] Bargonetti J, Champeil E, Tomasz M. 2010. Differential toxicity of DNA adducts of mitomycin C. J Nucleic Acids 2010:698960. doi:10.4061/2010/698960 20798760 PMC2925095

[51] Roske JJ, Liu S, Loll B, Neu U, Wahl MC. 2021. A skipping rope translocation mechanism in a widespread family of DNA repair helicases. Nucleic Acids Res 49:504–518. doi:10.1093/nar/gkaa1174 33300032 PMC7797055

[52] Burby PE, Simmons LA. 2019. A bacterial DNA repair pathway specific to a natural antibiotic. Mol Microbiol 111:338–353. doi:10.1111/mmi.14158 30379365 PMC6368877

