# Supplementary material for: Structural and functional investigation of DinG containing a 3′–5′ exonuclease domain
Source: mBio. 2025 Jun 30;16(8):e00884-25. doi: 10.1128/mbio.00884-25 (PMC12345164; doi:10.1128/mbio.00884-25)
Supplement: Supplemental material — Table S1 and Fig. S1 to Fig. S8. [file mbio.00884-25-s0001.pdf]

Structural and functional investigation of DinG containing a 3′–5′ exonuclease domain

Tianwen Gao[1]†, Wanshan Hao[1]†, Jin Gao[1]†, Yiyang Sun[1], Yukang Sun[1], Jieyu Yang[1], Kaiying Cheng[1,2]*

[1] Zhejiang Key Laboratory of Medical Epigenetics, Department of Immunology and Pathogen Biology, School of Basic Medical Sciences, Affiliated Hospital of Hangzhou Normal University, Hangzhou Normal University, Hangzhou, 311121, China.

[2] State Key Laboratory for Diagnosis and Treatment of Infectious Diseases, The First Affiliated Hospital, College of Medicine, Zhejiang University, Hangzhou, 310003, China.

†These authors have contributed equally to this work and share first authorship.

*To whom correspondence should be addressed. E-mail: kaiyingcheng@hznu.edu.cn

Supplementary table S1. Oligos used in this study.

(A) Primers used for cloning and mutagenesis.

Primers	Sequence (5′→3′)
DinG_up_F	tgatgaaagccaatccacctatgtcaaaatatagtcaagatgtac
DinG_up_R	tacgaaggatcctatttcacagctatctctccttgctttag
DinG_down_F	gtgaaataggatccttcgtagcgacattcaacagtttgg
DinG_down_R	tacttgctctcggacctattctccttcaatcatccagaac
pkor1_F	aataggtccgagagcaagtacgagcttaagactggcc
pkor1_R	aggtggattggctttcatcaatccccgtatagtgagtc
DinG_FN	ctgtattttcagggccatatgggtatggcaacctatg
DinG_RB	acggagctcgaattcggatcctcactttttcttttttgaatttgtc
DinG_D10A_F	tgcctgttgtttccaaagccacaacggcataggtt
DinG_D10A_R	aacctatgccgttgtggctttggaaacaacaggca
DinG_E12A_F	attggttgcctgttgttgccaaatccacaacggca
DinG_E12A_R	tgccgttgtggatttggcaacaacaggcaaccaat
DinG_H90A_F	caaatcaaaatctacattagcagcgacaaaaatgcaatc
DinG_H90A_R	gattgcatttttgtcgctgctaatgtagattttgatttg
DinG_D95A_F	ctttttaataaaattcaaagcaaaatctacattatgag
DinG_D95A_R	ctcataatgtagattttgctttgaattttattaaaaag
DinG_H149A_F	ggcatcttcgtcagctcgagcggcatttgctaaggtaata
DinG_H149A_R	tattaccttagcaaatgccgctcgagctgacgaagatgcc
DinG_D154A_F	gcagtagtagcggcagcttcgtcagctcgat
DinG_D154A_R	atcgagctgacgaagctgccgctactactgc

DinG_K336_338A_F	taaattttaaaattaatgcactattgattgcaagtgcaagtgattacatttcattgggac
DinG_K336_338A_R	gtcccaatgaaatgtaatcacttgcacttgcaatcaatagtgcattaattttaaaattta
DinG_H428_431A_F	aaattggtattacaaatcatgcagctttaattgcttctgatgttgaaaattcaatatatc
DinG_H428_431A_R	gatatattgaattttcaacatcagaagcaattaaagctgcatgatttgtaataccaattt
DinG_D448_449A_F	cgcaagtaaatatgctaatgatgcaccactgcctagtgatgcttca
DinG_D448_449A_R	tgaagcatcactaggcagtggtgcatcattagcatatttacttgcg
DinG_Y539A_F	gaagcgatgaatatcatcatcagcaacatcagaatcattgataatcg
DinG_Y539A_R	cgattatcaatgattctgatgttgctgatgatgatattcatcgcttc
DinG_K801A_F	ggtattaaatgtgtgatgatagcggcgttaccgtttatgaacaagcataat
DinG_K801A_R	attatgcttgttcataaacggtaacgccgctatcatcacacatttaatacc
DinG_F804A_F	tgtgtgatgatagcgaagttaccggctatgaacaagcataatgcaaaata
DinG_F804A_R	tattttgcattatgcttgttcatagccggtaacttcgctatcatcacaca
DinG_F823A_F	atggattctgaattcacttcaactgccaaggaatatgtattacctgatgc
DinG_F823A_R	gcatcaggtaatacatattccttggcagttgaagtgaattcagaatccat
DinG_R858A_F	cggcataatagtttcatttgatgatgcactcataaacagtaattataaaaac
DinG_R858A_R	gtttttataattactgtttatgagtgcatcatcaaatgaaactattatgccg
DinG_Y864A_F	gatgatcgactcataaacagtaatgctaaaaactttttgaacaaacacttg
DinG_Y864A_R	caagtgtttgttcaaaaagtttttagcattactgtttatgagtcgatcatc

(B) Oligos used for enzymatic assays.

**Name**	**Sequence (5′→3′)**
5nt dA	aaaaa
5nt dT	ttttt
5nt dG	ggggg
5nt dC	ccccc
5’FAM-20 nt poly dT	FAM-tttttttttttttttttttt
3’FAM-20 nt poly dT	tttttttttttttttttttt-FAM
Ja	agtaacgatggtgaaaggatgacagaagc-Dabsyl
Ja2	tctaagaccctgaaccggtagcaaaagtaacgatggtgaaaggatgacagaagc-Dabsyl
Jb	FAM-gcttctgtcatcctttcaccatcgttacttttgctaccgtcaatccatacttcg
anti-Jb	cgaagtatggattgacggtagcaaaagtaacgatggtgaaaggatgacagaagc-Dabsyl
Jb2	FAM-gcttctgtcatcctttcaccatcgttact
Jc3	cgaagtatggattgacggtagcaaaagtaacgatggtgagattctcaagatgac
Jd	gtcatcttgagaatctcaccatcgttacttttgctaccggttcagggtcttaga
Ja2m	tctaagaccctgaacaccgcagattagtaacgatggtgaaaggatgacagaagc
J98	FAMttctaagaccctgaaccactcgggaaataacaagatttcatctatgaccagtacgagcttaggtt gtcctggcccgcgtgcaaaggatgacagaagca
J98b	tgcttctgtcatcctttgcacgcgggccttttttttttttttttttttttttttttttttttttttttttttcccgagtggttc agggtcttagaa-Dabsyl
J98b2	cccgagtggttcagggtcttagaa-Dabsyl
J98u	FAM-ttctaagaccctgaaccactcgggaaa
J98d	ggcccgcgtgcaaaggatgacagaagca
anti-J98d	tgcttctgtcatcctttgcacgcgggcc

Supplementary figures

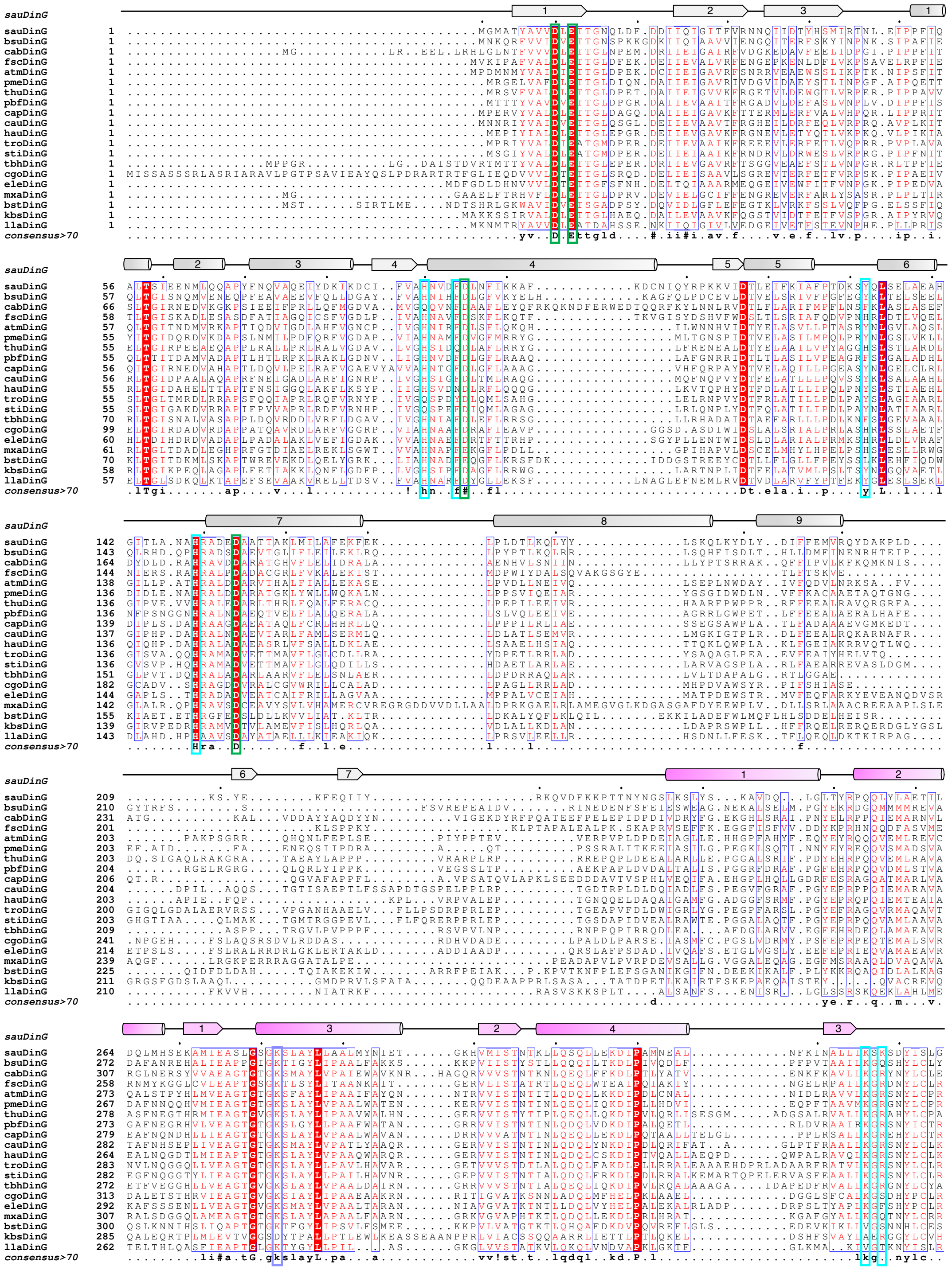

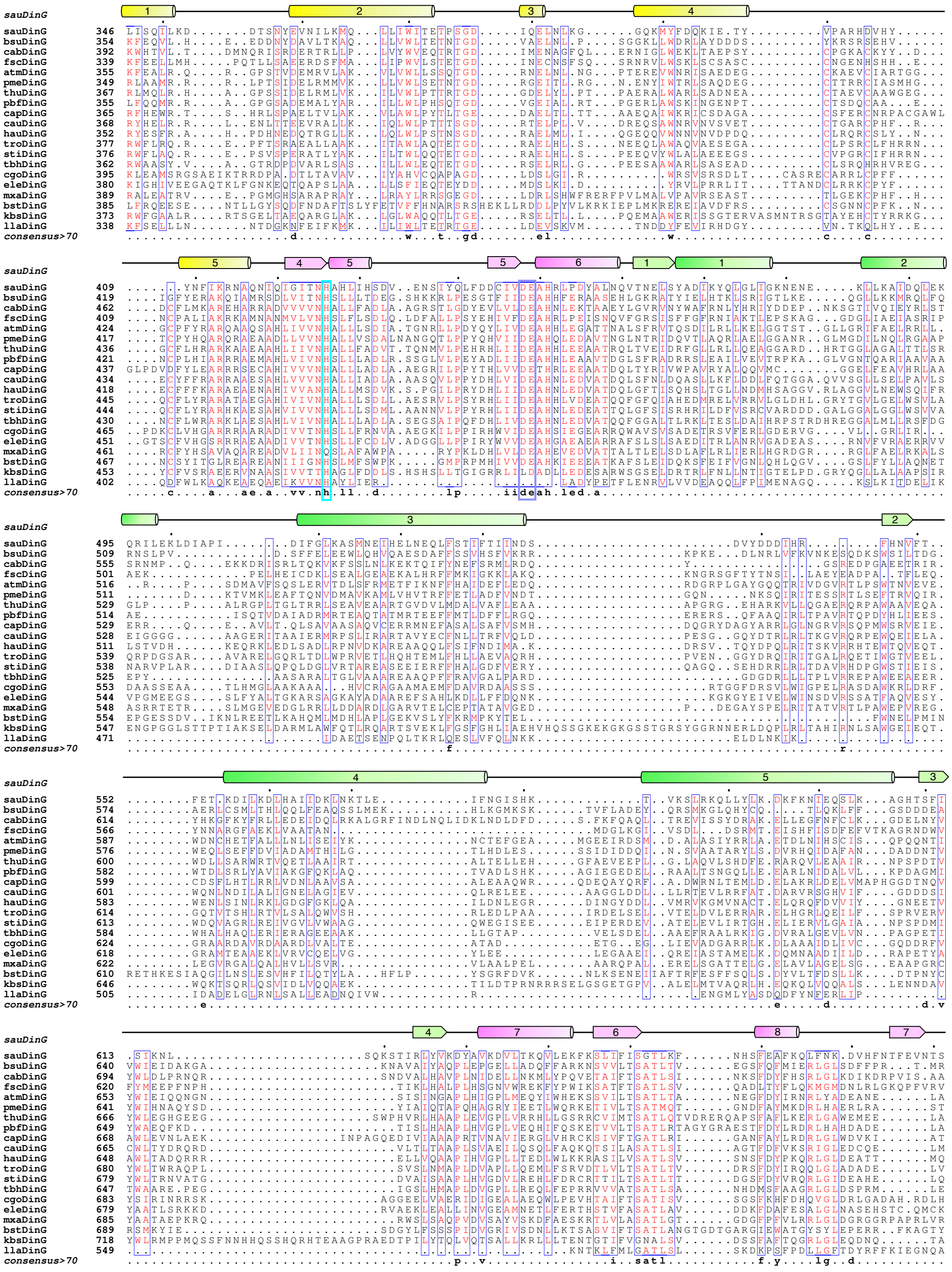
sauDinG
bsuDinG
cabDinG
fscDinG
atmDinG
pmeDinG
thuDinG
pbfDinG
capDinG
cauDinG
hauDinG
troDinG
stiDinG
tbhDinG
cgoDinG
eleDinG
mxaDinG
bstDinG
kbsDinG
llaDinG
consensus>70

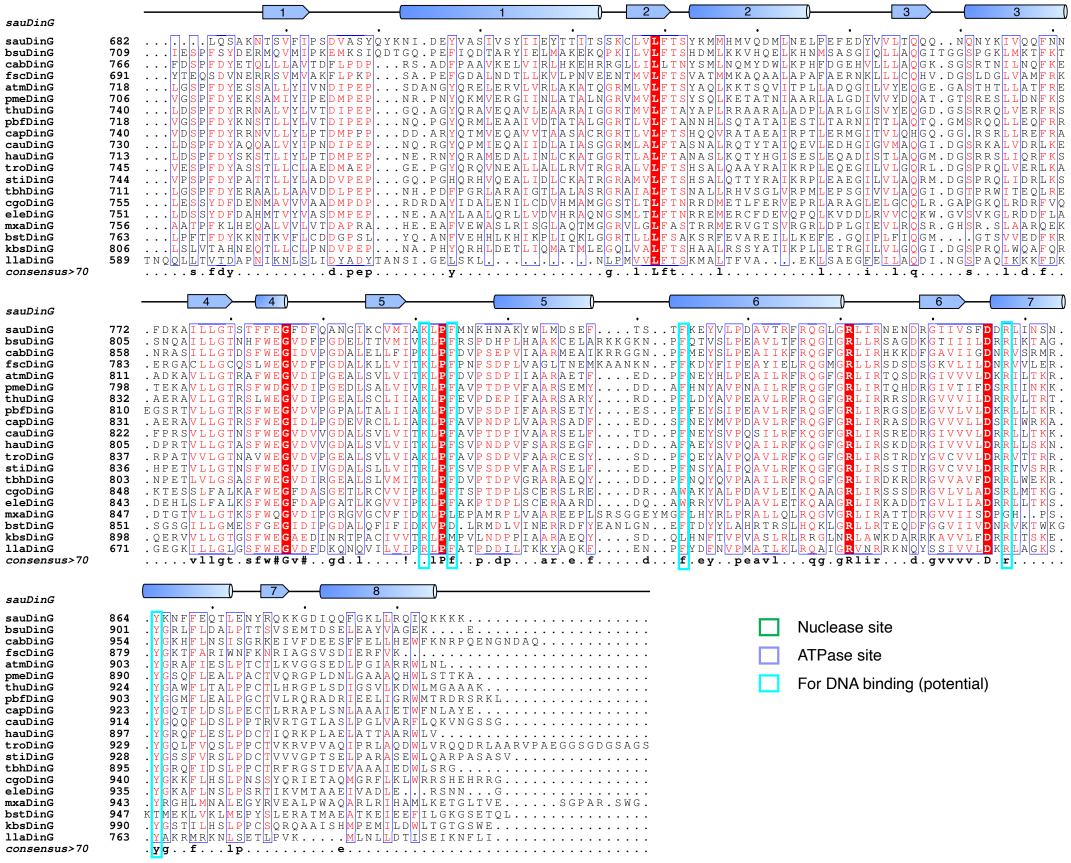

Supplementary figure S1. Sequence and structural analysis of DinGs containing the 3′–5′ exonuclease domain.

Sequence alignments of DinGs containing the 3′–5′ exonuclease domain from the representative species. sau, *S. aureus*; bsu, *Bacillus subtilis*; cab, *Caldithrix abyssi*; fsc, *Fibrobacter succinogenes*; atm, *Anaerolinea thermophila*; pme, *Phototrophicus methaneseepsis*; thu, *Thermoflexus hugenholtzii*; pbf, *Candidatus Promineofilum breve*; cap, *Caldilinea aerophile*; cau, *Chloroflexus aurantiacus*; hau, *Herpetosiphon aurantiacus*; tro, *Thermomicrobium roseum*; sti, *Sphaerobacter thermophilus*; tbh, *Tepidiforma bonchosmolovskayae*; cgo, *Coriobacterium glomerans*; ele, *Eggerthella lenta*; mxa, *Myxococcus xanthus*; bst, *Bacteriovorax stolpii*; kbs, *Ktedonobacterales bacterium* SCAWS-G2; lla, *Lactococcus lactis* subsp. lactis Il1403; Secondary structural elements are depicted based on the SaDinG structure solved in this study, shown at the top of the sequences. The critical residues for catalysis and for DNA binding were highlighted in green and cyan boxes, respectively.

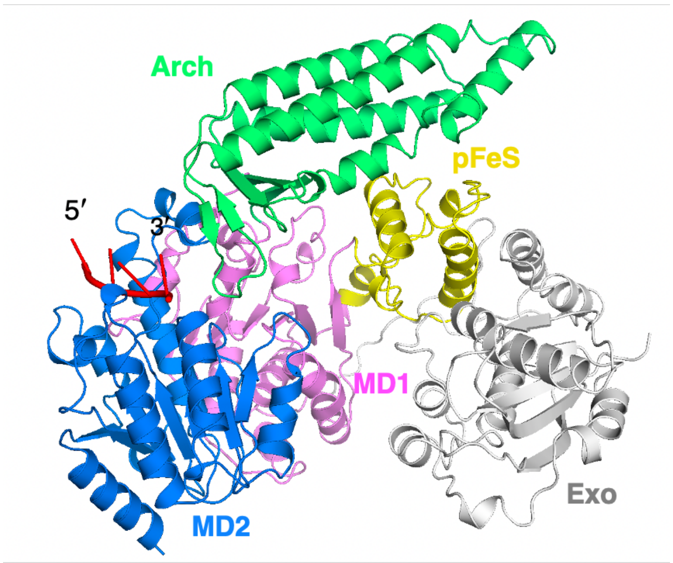

Supplementary figure S2. The overall structure of SaDinG-ssDNA complex

The SaDinG-ssDNA complex structure was depicted in a cartoon. The 3′–5′ Exo, MD1, MD2, Arch, and pFeS domains were colored white, violet, marine, lime green, and yellow, respectively. ssDNA was colored red.

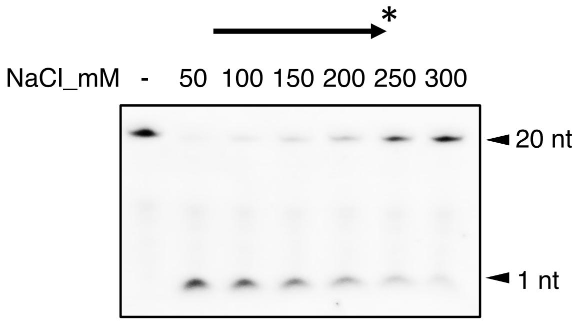

Supplementary figure S3. The optimum salt concentration test of SaDinG.

100 nM 3' FAM labeled 20 nt poly dT was incubated with 0.1 μM SaDinG, in the presence of 10 mM Mg^{2+} and varying concentrations of NaCl (0, 50, 100, 150, 200, 250, and 300 mM), and the resulting products were separated on a denaturing PAGE gel.

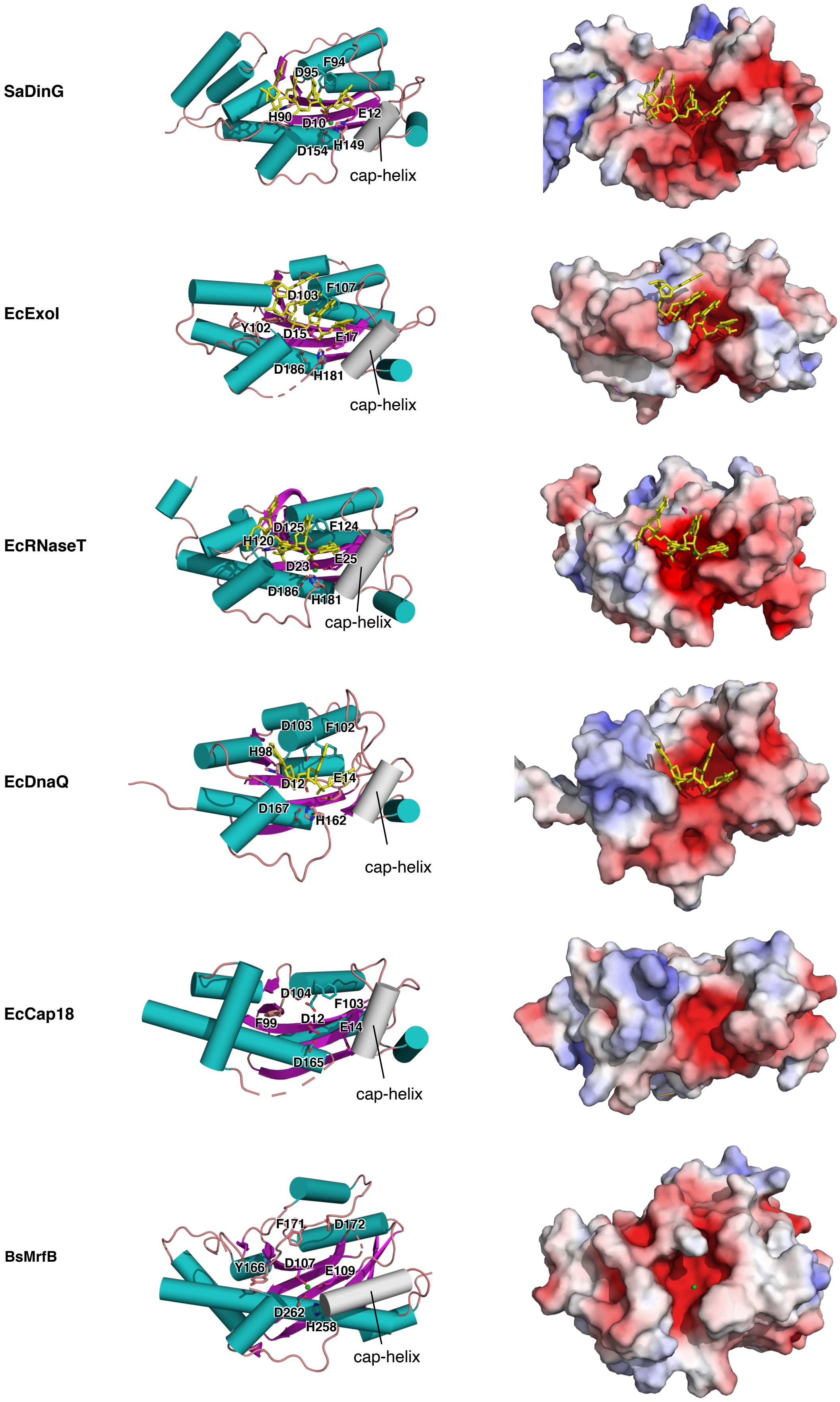
SaDinG
F94
D95
H90
D10
E12
H149
D154
cap-helix
EcExoI
D103
F107
Y102
D15
E17
D186
H181
cap-helix
EcRNaseT
D125
F124
H120
D23
E25
D186
H181
cap-helix
EcDnaQ
D103
F102
H98
D12
E14
D167
H162
cap-helix
EcCap18
D104
F103
F99
D12
E14
D165
cap-helix
BsMrfB
F171
D172
Y166
D107
E109
D262
H258
cap-helix

Supplementary figure S4. Comparisons of the exonuclease domain of different DEDDh-type 3'-5' exonuclease.

The zoom in view of the exonuclease domains from SaDinG (PDB code: 8ZEF), *E. coli* Exonuclease I (EcExoI, PDB code: 4JS4), *E. coli* RNase T (EcRNaseT, PDB code: 3V9X), *E. coli* DnaQ (EcDnaQ, PDB code: 5M1S), *E. coli* Cap18 (EcCap18, PDB code: 7T2S), and *B. subtilis* MrfB (BsMrfB, PDB code: 8UN9), were shown as cartoon (left) and their electrostatic potentials were determined using APBS, which were then projected onto the solvent-accessible surface of the structure at contouring levels of ±5 kT (right, depicted in blue/red). The key residues involved in metal ion coordination and substrate interaction were shown as sticks. DNA bases were represented as sticks, coloued yellow. The cap-helices were illustrated white.

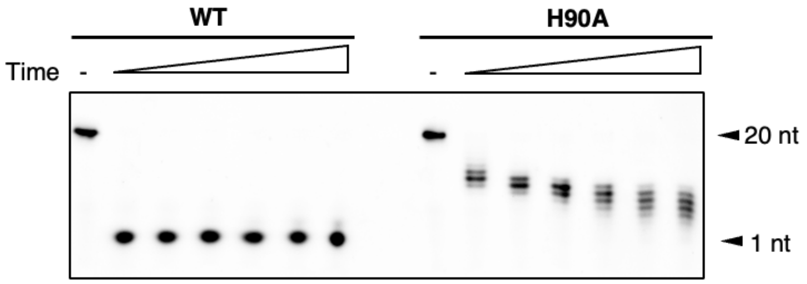

Supplementary figure S5. Comparison of the digestion efficiency between wild-type and H90A mutant.

Reactions contained 100 nM 5'-FAM-labeled 20-nt poly(dT) substrate, 50 nM wild-type SaDinG and H90A mutant, and 1 mM Mn^{2+}, incubated for indicated time points (0, 2, 5, 10, 30, 60, and 90 min). Products were analyzed by denaturing PAGE.

A

B

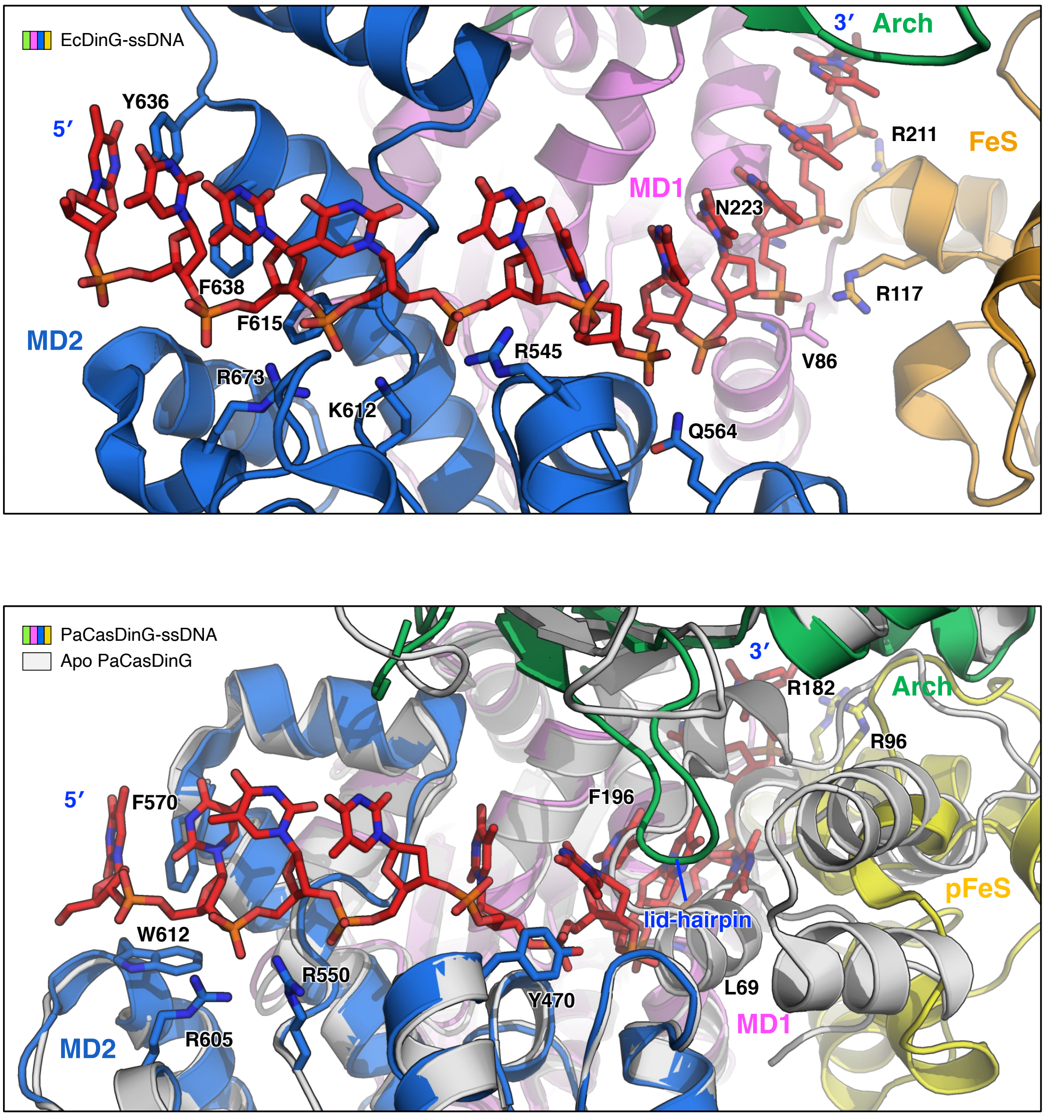

Supplementary figure S6. The DNA binding channel of EcDinG and PaCasDinG. (A) The cartoon view of the DNA binding channel of EcDinG-ssDNA complex (PDB code:6FWR). (B) The cartoon view of the DNA binding channel of PaCasDinG-ssDNA complex (PDB code: 7XF1) and apo PaCasDinG (PDB code: 7XEX). The PaCasDinG-ssDNA complex was coloured by domains, consistent with SaDinG, while the apo PaCasDinG structure was shown in white-grey. Key residues involved in DNA binding were shown as sticks and labelled.

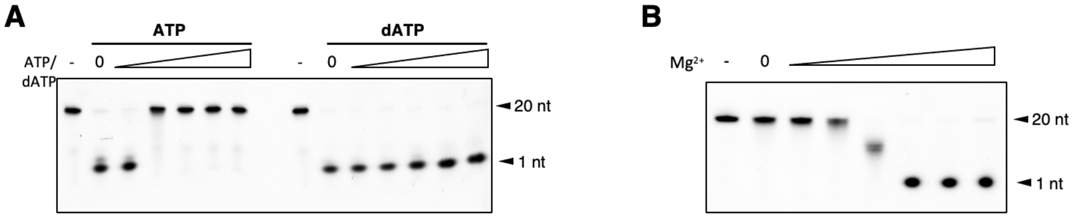

Supplementary figure S7. The inhibition effect of ATP on the nuclease activity of SaDinG

(A) Effects of different concentrations of ATP and dATP on the nuclease activity of SaDinG. A reaction mixture containing 100 nM 5'-FAM-labeled 20 nt poly(dT) and 50 nM SaDinG was incubated in the presence of 10 mM Mg^{2+} and varying concentrations of ATP or dATP (0, 0.625, 1.25, 2.5, 5, or 10 mM). The resulting products were resolved by denaturing PAGE. The lane marked "-" represents the control reaction without ATP or Mg^{2+}. (B) Effects of Mg^{2+} concentration on SaDinG nuclease activity in the presence of ATP. A reaction mixture containing 100 nM 5'-FAM-labeled 20 nt poly(dT) and 50 nM SaDinG was incubated with 0.625 mM ATP and varying concentrations of $MgCl_2$ (0, 0.625, 1.25, 2.5, 5, 10, or 20 mM). The resulting products were resolved by denaturing PAGE. The lane marked "-" represents the control reaction without ATP or Mg^{2+}.

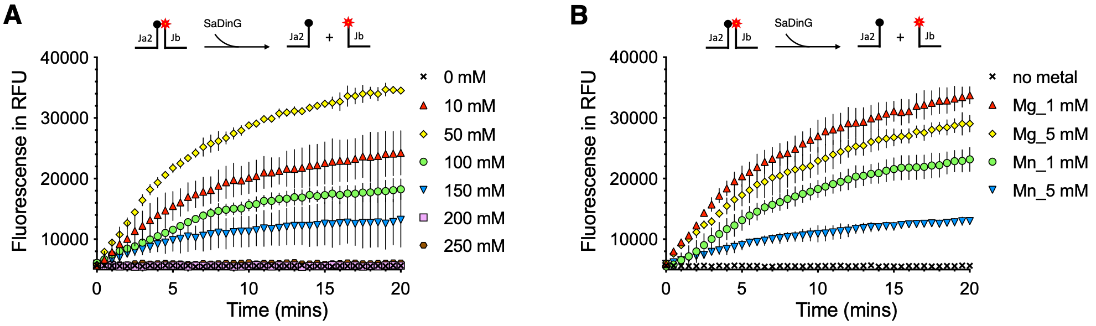

Supplementary figure S8. The helicase activity of SaDinG.

(A) Real-time unwinding assays to test the optimum salt concentration. The real-time unwound fractions of splayed duplex were monitored at different NaCl concentrations (0, 10, 50, 100, 150, 200, and 250 mM), and shown as curves. 25 nM substrate was incubated with 0.5 µM nuclease dead SaDinG (D10A), 1 mM ATP, and 1 mM $MgCl_2$. Each assay was repeated three time. (B) Real-time unwinding assays to test the optimum metal concentration. The real-time unwound fractions of splayed duplex were monitored at different $MgCl_2$ or $MnCl_2$ concentrations (0, 1, and 5 mM), and shown as curves. 25 nM substrate was incubated with 0.5 µM nuclease dead SaDinG (D10A), and 1 mM ATP, and a specific concertation of metal ions. Each assay was repeated three times.
